# A model of binding on DNA microarrays: understanding the combined effect of probe synthesis failure, cross-hybridization, DNA fragmentation and other experimental details of affymetrix arrays

**DOI:** 10.1186/1471-2164-13-737

**Published:** 2012-12-27

**Authors:** Yasminka A Jakubek, David J Cutler

**Affiliations:** 1 Department of Human Genetics, Emory University School of Medicine, Atlanta, GA, 30322, USA; 2 Graduate Program in Genetics and Molecular Biology, Emory University, Atlanta, GA, 30322, USA

## Abstract

**Background:**

DNA microarrays are used both for research and for diagnostics. In research, Affymetrix arrays are commonly used for genome wide association studies, resequencing, and for gene expression analysis. These arrays provide large amounts of data. This data is analyzed using statistical methods that quite often discard a large portion of the information. Most of the information that is lost comes from probes that systematically fail across chips and from batch effects. The aim of this study was to develop a comprehensive model for hybridization that predicts probe intensities for Affymetrix arrays and that could provide a basis for improved microarray analysis and probe development. The first part of the model calculates probe binding affinities to all the possible targets in the hybridization solution using the Langmuir isotherm. In the second part of the model we integrate details that are specific to each experiment and contribute to the differences between hybridization in solution and on the microarray. These details include fragmentation, wash stringency, temperature, salt concentration, and scanner settings. Furthermore, the model fits probe synthesis efficiency and target concentration parameters directly to the data. All the parameters used in the model have a well-established physical origin.

**Results:**

For the 302 chips that were analyzed the mean correlation between expected and observed probe intensities was 0.701 with a range of 0.88 to 0.55. All available chips were included in the analysis regardless of the data quality. Our results show that batch effects arise from differences in probe synthesis, scanner settings, wash strength, and target fragmentation. We also show that probe synthesis efficiencies for different nucleotides are not uniform.

**Conclusions:**

To date this is the most complete model for binding on microarrays. This is the first model that includes both probe synthesis efficiency and hybridization kinetics/cross-hybridization. These two factors are sequence dependent and have a large impact on probe intensity. The results presented here provide novel insight into the effect of probe synthesis errors on Affymetrix microarrays; furthermore, the algorithms developed in this work provide useful tools for the analysis of cross-hybridization, probe synthesis efficiency, fragmentation, wash stringency, temperature, and salt concentration on microarray intensities.

## Background

DNA microarray chips consist of large numbers of probes, single stranded DNA molecules attached to a solid surface, that hybridize to nucleic acids [1]. Microarrays have several uses in DNA analysis including CNV detection [2-5], re-sequencing [6], SNP typing [7,8] , detection of species specific DNA in complex samples [1], and identification of protein-DNA binding sites [1]. They are also used to assess transcript levels in samples of coding and non-coding RNA [1,9,10]. In the field of human genetics, DNA microarrays are used to investigate disease [4,11,12], to study variation [5,8], and to detect variants in clinical samples [9].

Collections of identical probes are called probe spots or features. Each probe spot consists of many copies of identical single stranded DNA molecules. Many DNA array designs have multiple features querying the same target DNA. Often one set of features queries targets on the forward strand of the DNA while the other set queries targets on the reverse strand. The first step in a DNA microarray experiments is to isolate and amplify the target DNA or RNA. Next, the amplified target is fragmented and fluorescently labeled. The labeled target solution is then hybridized to the chip where target binds to probe DNA. Following hybridization the chip is washed in order to eliminate non-specific binding. Finally, the chip is scanned and the fluorescent intensity measured for each feature.

The fundamental assumption behind a DNA microarray experiment is that the intensity measure for a probe spot correlates to the concentration of target bound to that spot, which in turn correlates to the amount of target in the original solution [6,13]. However, the relationship between observed intensity and target DNA composition is not straightforward. Known variables with microarrays include high variance in intensity between probe spots, high variance in a single probe spot’s intensity between chips, as well as background (non-specific binding) intensity differences between chips. Several studies have focused on the binding kinetics of DNA molecules attached to a solid surface and on cross-hybridization [14-18]. These studies have helped illuminate some aspects of the microarray experiment; however, several fundamental observations of microarray behavior remain poorly understood. First, when an array contains probe spots for both the forward and reverse target, simple liquid phase kinetics predict that both probe spots ought to have the same binding affinities and therefore should have equal amounts of bound target DNA [19]. However, in practice forward and reverse strand probe spots usually have significantly different intensity measurements [20]. These differences in intensity are observed in both GC and AT rich probes as well as probes with and without nucleotide runs (20). Second, liquid phase kinetics predicts that mismatches anywhere in the oligo (other than in the last 3 bases) ought to have equal effects on binding [19,21-25]. However, mismatches near the center of the probe have a stronger effect on probe intensity compared to mismatches towards the edges [26]. Third, chips manufactured on different days often have subtly different binding properties (so-called chip-effects); as do chips processed by different facilities or on different days (batch effects) [27,28].

The goal of this study is to attempt to understand all of these aspects. To do so we develop a detailed model of the DNA microarray experiment and then use this model to predict probe intensities for seven different microarray designs.

The basic assumption behind this modeling approach is that the hybridization kinetics of DNA binding on a chip are fundamentally the same as liquid phase kinetics. Apparent differences between liquid phase predictions and microarray observations arise from the combined effect of different aspects of the microarray experiment. In the model we include the effect of probe sequence, cross-hybridization, nucleotide position, and hybridization conditions. We model the combined effect of these factors in one step rather than normalizing the data for each factor in a stepwise manner [29,30]. Unlike previous studies we do not adjust binding strength for nucleotides based on their position on the probe [14]. Instead the “positional” effect of nucleotides arises naturally in our model as a side effect of target DNA fragmentation and microarray synthesis. In particular we assume that microarray synthesis is not perfect [31-33]; more specifically, we assume that during probe synthesis individual A, C, G, and T nucleotides fail to incorporate at different rates. We also model abasic sites on the probe sequence where the probabilities that A, C, G, and T nuclotides become abasic are not necessarily equal to each other. Consequently differences in synthesis efficiency/abasic sites between nucleotides explain why forward and reverse DNA probe spots often have significantly different intensities and explain chip-effects. Additionally we explicitly model target DNA concentration, hybridization temperature, mean fragmentation size, wash stringency [15,34,35], and microarray scanner settings [36] which together with errors along the probe sequence give rise to batch effects. Previous studies have reported that different probe sequences have different saturation intensities [34], under our model this is expected given that each probe spot consists of a “forest” of probes and that the number of probes capable of binding target DNA is sequence dependent. Furthermore, the strength of the wash impacts the final number of probe/target duplexes [34].

Our model consists of two parts. First, we calculate binding affinities for the probes and target DNA in the hybridization solution. To do so we use the Langmuir isotherm. This part of the model is independent from the chip intensity data and simply yields equilibrium constants for all possible target-probe complexes. In the second part of the model the binding data is used to predict probe intensity for Affymetrix arrays. In this step, we fit several parameters (probe synthesis efficiency, wash stringency, fragmentation, scanner’s dynamic range, and target DNA concentration) to each individual chip and predict the probe intensities for that chip. For the analyzed data the average correlations ranged from 0.88 and 0.55. Our results show that the different bases (A, C, G, and T) do not incorporate into the probe with the same efficiency.

## Methods

Our model begins with the assumption that DNA hybridization on a microarray is the same as DNA hybridization in solution, but that many of the experimental details previously ignored as well as other details of the microarray experiments must be explicitly modeled to account for the observed differences between solution and microarray. In particular, we model target fragmentation, cross-hybridization, microarray synthesis imperfections, the effect of the wash, and the scanner’s dynamic range. In the final model we must fit at least ten parameters specific to each microarray (eight parameters related to synthesis efficiencies, one parameter for the mean fragmentation size of the target, and one concentration parameter per target molecule).The model also includes four parameters that vary between batches of microarrays processed at the same time (one parameter for the wash common between chips, and three parameters related to the shape of the scanner’s dynamic range).

### K_eq_ calculations

Our approach to target DNA/probe DNA hybridization is exhaustive. We begin by assuming that hybridization temperature, hybridization solution salt concentration, probe sequences, and target DNA sequences are known. In our model the target DNA consists of one or more DNA “segments” which have unknown concentrations. These DNA “segments” are user defined and can be PCR fragments, reduced representations of the genome, chromosomes, transcripts, whole genomes, or any other set of DNA sequences that accurately represents the segments produced by the experimental protocol. First we fragment the target at every possible position; thus, modeling all potential cut sites on the target DNA. We then allow each of the resulting target fragments to bind to every position on every probe. Thus in our model, a probe spot consists of a “forest” of bound target/probe complexes. The target molecules that are hybridized to the probes in a given probe spot are of differing lengths and are bound at differing start and end-positions of the probe sequence. Even though each spot consists of a complex assortment of probe/target complexes the underlying thermodynamics of each individual binding reaction is fundamentally the same as in solution and follows a Langmuir isotherm with nearest-neighbor kinetics [19,37,38].

**Figure 1:**
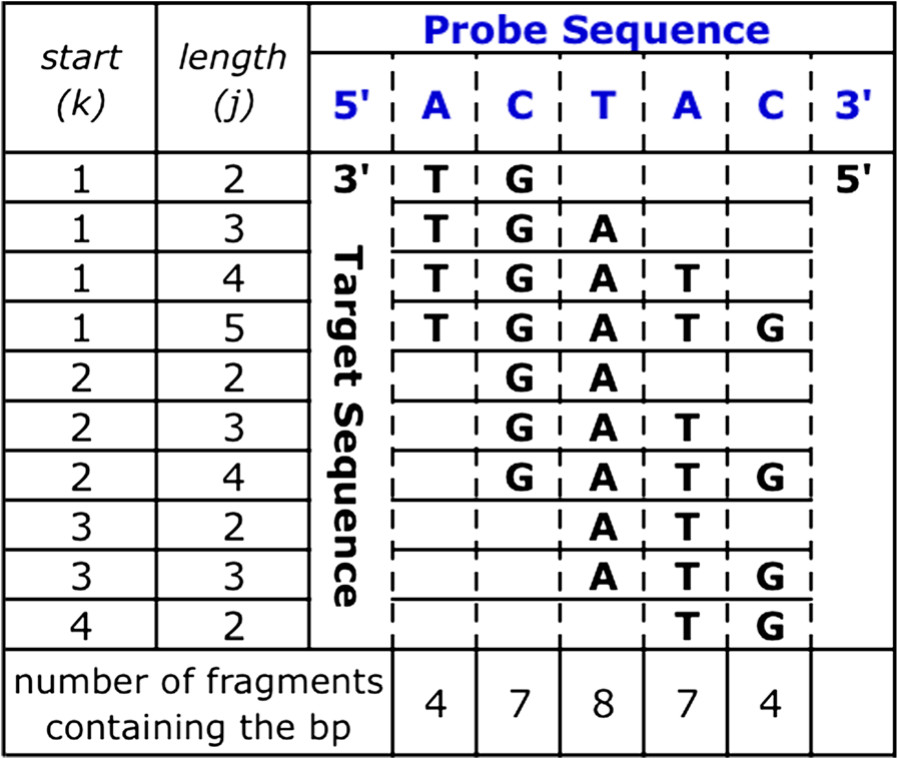
**Probe and Target Sequences.** Sample probe sequence in blue. All unique target sequences that are 2 base pairs or longer and that are perfect reverse complements of the probe in black. Columns 1 and 2 have the corresponding k and j values for each target sequence. The bottom row counts the number of times each probe position binds to a different target fragment.

**Figure 2:**
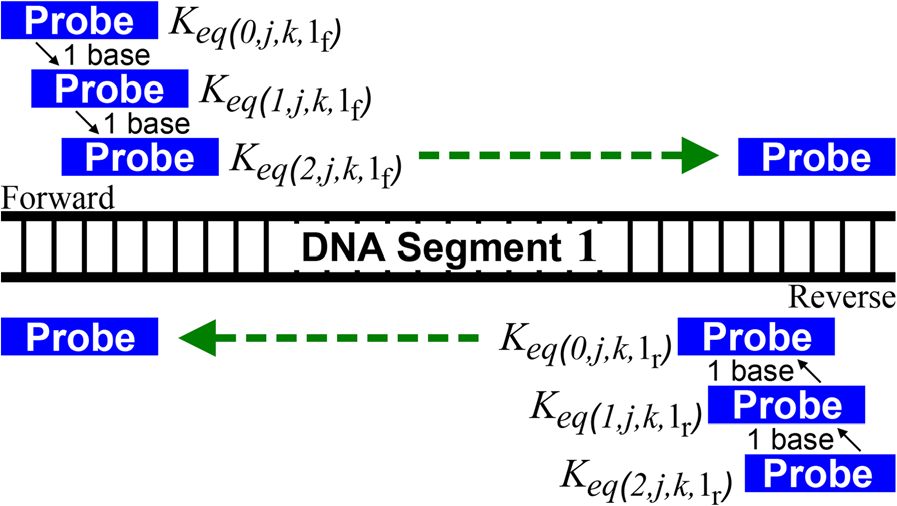
**Probe-Target Binding.** Ways in which a probe sequence is aligned to the target DNA. Only one target DNA segment is shown. The probe is aligned to both the forward and reverse target sequences along all positions (i) of the target DNA and for all appropriate j and k values.

In order to model target binding along all positions of a probe, we split the target into all possible sequences that are ≥2 base pairs (bp) (Figure 1). For each of these sequences we calculate the K_eq_ (equilibrium constant) values for the forward and reverse target sequences aligned to every position (Figure 2) of the probe. To calculate each K_eq_ value we first calculate the change in free energy, ΔG, for each of these probe-DNA duplexes. Where ΔG is

(1) △G=△H−T△S

ΔH is change in enthalpy, ΔS is change in entropy, and T is hybridization temperature. We calculate ΔG using the nearest neighbor model [37,38]. The values for ΔH, and ΔS for perfect match and mismatch base pairs, initiation/termination GC and AT values, and [Na+] corrections for ΔG calculations come from [19,21-25]. All the nearest neighbor values used to calculate changes in free energy are listed in the supplementary materials (Additional file 1: Table S1A). We then calculate using

(2) $$K_{\text{eq}}=e^{\frac{-△G}{\text{RT}}}$$

where R is the gas constant.

### Fraction bound probes calculations

After we calculate target DNA/probe DNA binding thermodynamics, for all possible target/probe combinations we use those K_eq_ values to calculate the fraction of bound probes, α, for each probe spot. To do so we assume the Langmuir isotherm

(3) $$CK_{\text{eq}}=\frac{\alpha }{1-\alpha }$$

where C is equal to the target DNA concentration. Rearranging, we solve for α and get

(4) $$\alpha =\frac{CK_{\text{eq}}}{CK_{\text{eq}}+1}$$

In order to use this formula for α, two conditions have to be met. First, equilibrium for target/probe formation must be reached, and second, [C] >> [probe], such that Δ[C] due to target/probe binding is negligible.

Equation 4 holds for a single target sequence and a single probe spot. However, the experiment consists of long segments of DNA fragmented at random into a collection of targets of differing lengths with differing start and stop positions. Our first assumption is that the fragmentation process creates a uniform pattern of fragmentation, such that the probability that the target is “cut” between any two bases is equal. Thus, start and stop positions of every target DNA fragment are uniformly distributed, and the lengths of the fragments in between the cut sites are geometrically distributed. We use the mean fragment size, μ, estimated from the data, to calculate p, the probability that the target is fragmented between any given pair of bases, and q = 1-p. Let C_i,j_ be the concentration of target with a cut after the i^th^ base, and another cut j bases later. The concentration of such fragment (C_i,j_) is

(5) $$C_{i,j}=C*p^{2}q^{j-1}$$

where C is the overall concentration of the target molecule. For target molecules with a cut after base i, and extending at least j bases without a cut, but which continues an unknown number of bases past the end of the probe, we estimate the concentration as

(6) $$C_{i}=\sum_{k=j}^{\infty }Cp^{2}q^{k}=\text{Cp}q^{j}$$

Each of the C_i,j_ fragments can bind at any position in a probe. Let K_eq__(i,j,k)_ equal to the K_eq_ when fragment C_i,j_ binds starting at position k of the probe. For any one fragment (i,j) bound at a position k, the fraction of bound probes α_(i,j,k)_ is

(7) $$\alpha_{i,j,k}=\frac{C_{i,j,k}K_{\text{eq}\left(i,j,k\right)}}{C_{i,j,k}K_{\text{eq}\left(i,j,k\right)}+1}$$

Equation 7 fails to model competitive hybridization. When we incorporate competition between targets we must sum all possible fragment and target duplexes [39] to get

(8) $$\alpha =\frac{\sum_{k}\sum_{i}\sum_{j}C_{i,j,k}K_{\text{eq}\left(i,j,k\right)}}{\left(\sum_{k}\sum_{i}\sum_{j}C_{i,j,k}K_{\text{eq}\left(i,j,k\right)}\right)+1}$$

When the target solution consists of n distinct DNA segments with different concentrations the fraction of bound probes for a given probe spot becomes

(9) $$\alpha =\frac{\sum_{n}\sum_{k}\sum_{i}\sum_{j}C_{i,j,k,n}K_{\text{eq}\left(i,j,k,n\right)}}{\left(\sum_{n}\sum_{k}\sum_{i}\sum_{j}C_{i,j,k,n}K_{\text{eq}\left(i,j,k,n\right)}\right)+1}$$

where C_n_ is equal to the concentration of the n^th^ DNA segment.

In our model competitive hybridization only takes place during the hybridization period. Following this hybridization period, the chip is washed with a low salt solution. We model the wash as a K_eq_ threshold. We assume that any probe-target complexes with K_eq_ values below this threshold come apart and no new target/probe complexes form during the wash period. We modify Formula 9 above to include the effect of the wash by excluding K_eq_ values < K_eqW_ threshold values in the numerator term. To do so we modify α

(10) $$\alpha =\frac{\sum_{n}\sum_{k}\sum_{i}\sum_{j}C_{i,j,k,n}K_{\mathrm{eqW}\left(i,j,k,n\right)}}{\left(\sum_{n}\sum_{k}\sum_{i}\sum_{j}C_{i,j,k,n}K_{\text{eq}\left(i,j,k,n\right)}\right)+1}$$

where K_eqW(i,j,k)_ are all K_eq_ values greater than K_eqW_.

Equation 10 above describes probe/target binding when there are no errors during probe synthesis and consequently all oligos in a probe spot are identical in sequence and in length. However, errors in probe manufacturing are common [31-33] and will alter probe binding efficiencies. In our model we employ two distinct mechanisms of error: “truncation errors” and “abasic” sites (Table 1). We define truncation errors as the failure of a nucleotide to incorporate during the protection/deprotection stage of probe synthesis; consequently these errors cause the probe to be truncated at a given spot, with no further incorporation of bases. A site is said to be abasic, if the DNA backbone is present, but the nucleotide is not. Sites are assumed to become abasic some time after probe synthesis, but prior to hybridization. Due to both of these types of errors, probe spots consist of a heterogeneous mixture of probes. These spots have full-length probes (no errors) as well as probes with one or more errors (Table 1). Since probe synthesis starts at the 3’ end, a probe with a truncation error will extend from the 3’ end up to the last base that was successfully incorporated. Probes with abasic sites, on the other hand, vary from the full length probe only at the site where the nucleotide was lost. We further assume that the probability of an error is independent and identically distributed and fixed across a microarray. Let *A*_s_, *C*_*s*_*, G*_*s*_, and *T*_*s*_ be the probabilities that A, C, G and T nucleotides are synthesized correctly (contain no truncation error) and A_B_, C_B_, G_B_, and T_B_ be the probabilities that A, C, G, and T nucleotides do not subsequently lose their base. The probability that a probe is full length p_F_ (no errors) is

(11) $$p_{F}=A_{S}^{\text{nA}}C_{S}^{\text{nC}}G_{S}^{\text{nG}}T_{S}^{\text{nT}}A_{B}^{\text{nA}}C_{B}^{\text{nC}}G_{B}^{\text{nG}}T_{B}^{\text{nT}}$$

where nA, nC, nG, and nT are the number of A, C, G, and T bases in the full length probe. Similarly the probability that a probe has exactly one abasic site at an A nucleotide is equal to

(12) $$\frac{p_{F}\left(1-A_{B}\right)}{A_{B}}$$

**Table 1:** Synthesis errors

**Probe sequence**	**Type of synthesis error(s)**
5’ C^12^T^11^A^10^C^9^C^8^G^7^T^6^A^5^C^4^C^3^G^2^T^1^ 3’	Full length probe (no error)
5’ C^8^G^7^T^6^A^5^C^4^C^3^G^2^T^1^ 3’	Incorporation error base 9
5’ C^8^G^7^T^6^_^5^C^4^C^3^G^2^T^1^ 3’	Incorporation error base 9 and abasic site
5’ C^12^T^11^A^10^_^9^C^8^G^7^T^6^_^5^C^4^C^3^G^2^T^1^ 3’	Full length probe with two abasic sites
5’ T^6^_^5^C^4^_^3^G^2^T^1^ 3’	Incorporation error base 7 and two abasic sites

Table 1: (_) denotes an abasic site.

We calculate all other one error probabilities in a similar manner.

When we account for synthesis failure, we imagine the spot as composed of a forest of full length probes together with all possible manufacturing errors for that probe. We calculate α_x_ for each possible error, x, individually, and create an overall α by weighting each by the probability, p_x_, that this particular error will occur. Thus,

(13) $$\alpha =p_{F}\alpha_{F}+\sum_{x=1}^{l}p_{x}\alpha_{x}$$

where the sums are taken over all possible errors x. To simplify calculations, we assume that probes with two or more errors have little to no binding and an α_x_ value of zero.

### Estimating probe spot intensity

**Figure 3:**
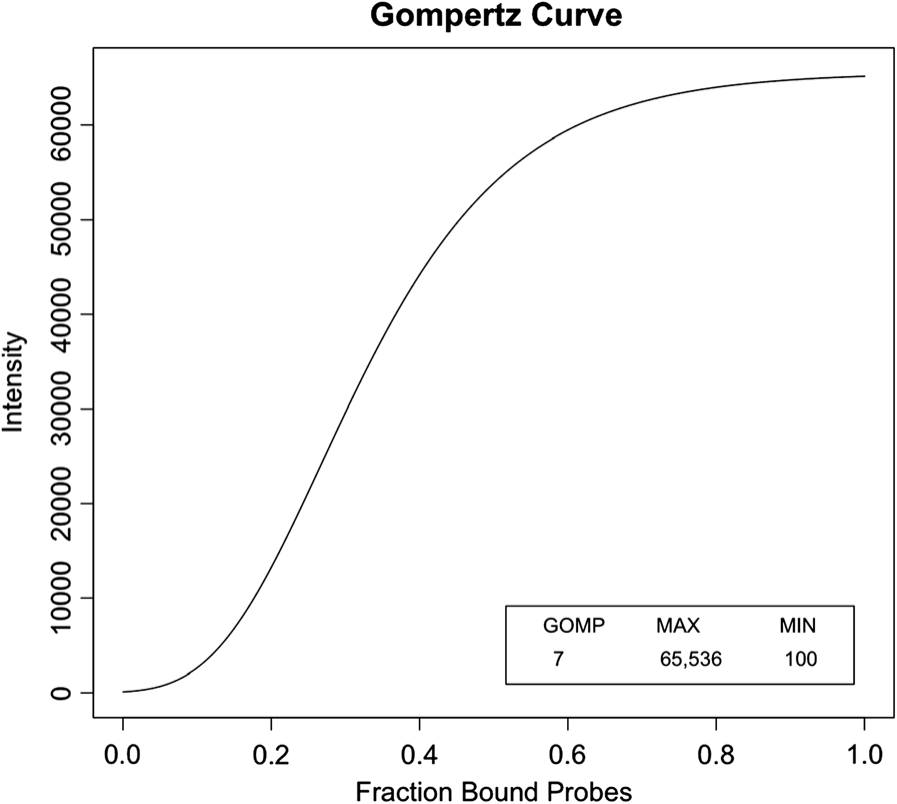
**Gompertz Curve.** Sample Gompertz Curve.

Equation 13 gives us the proportion of probes bound. By assumption, probes with more target bound will have greater florescent intensity when the probe is scanned. In general, the aim is to have a nearly linear relationship between the amount of probe bound and the observed florescent intensity. However, it is absolutely certain that at the limits of the scanner’s dynamic range, a linear response is physically impossible [36]. In order to model this and other factors such as quenching and the dynamic range of the a/d converter, we assume that the relationship between observed florescent intensity and the proportion of probes bound follows a gompertz curve and the expected intensity is equal to

(14) $$\text{MAX}*e^{\left(\text{log}\left(\frac{\text{MIN}}{\text{MAX}}\right)*\left(e^{-\alpha *\text{GOMP}}\right)\right)}$$

where MIN is equal to the background intensity, MAX is equal to the linear cutoff for intensity, and GOMP is equal to the shape parameter of the gompertz curve (Figure 3). The user supplies the MIN, MAX, and GOMP values.

We fit four parameters for the truncation rates, four parameters for the rate of abasic sites, and a variable number of parameters for the target DNA segment concentrations. To do so we minimize the square difference between the observed and expected probe intensities across all probe spots on a chip, using Powell’s method for numerical minimization [40]. In order to ensure that the algorithm did not find a sub-optimal solution, we ran several searches on the same chip. Each time the algorithm would start at a different part of parameter space and it always found the same solution.

### One dimensional nearest neighbor and initiation termination parameter search

We performed a one-dimensional search for the NN and the initiation/termination parameters used in the K_eq_ calculations. In this search we maximized the observed mean correlation for a set of five chips each ran under four different combinations of wash/fragmentation parameter values.

### The effect of mismatches on probe intensities

To understand the effect of mismatches under our model we compared the intensity of “perfect match” probes to all possible “mismatched probes.” More specifically we calculated the expected intensity of 20,102 probes that align perfectly to the human reference sequence. Then for each of these 25 bp long probes we calculated the expected intensity for all sequences that have all possible one base pair differences. Therefore, for each “perfect match” probe there are 75 “mismatch” probes. The “perfect match” probe sequences come from the FMR1 chip design and the parameters used to calculate the expected intensities are the same as the parameters that were fit for chip number 19 FMR1 design (Additional file 1: Table S2).

### Comparison of forward and reverse strand probe intensities

We compared the observed and expected intensities for probes that perfectly matched the reference sequence. For every forward and reverse probe pair we calculated the log ratio of the forward/reverse observed intensities. We then calculated the average of this ratio across all 62 chips from the FMR1 design. We did the same for the expected intensity. Then for each probe pair we estimated the difference in nucleotide composition between forward and reverse as the sum of (A-T) and (G-C). Where A, C, G, and T are the number of A, C, G, and T bases in the forward strand probe. We used this value to create bins for the means of the ratios. We then plotted the mean for the observed and expected ratios for each bin (Additional file 2: Figure S1).

## Results

**Figure 4:**
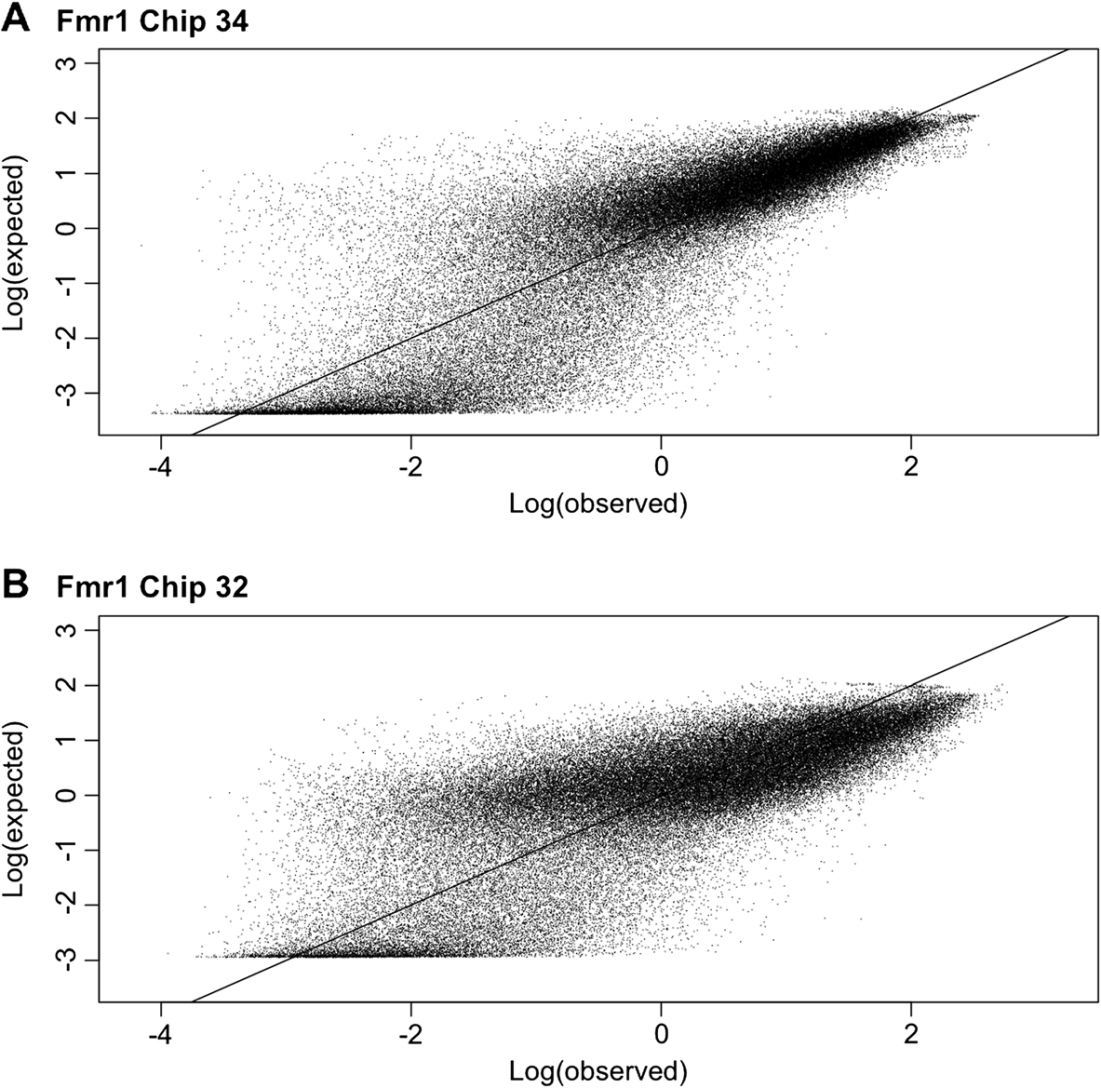
**Observed and Expected Intensity Plots.** Plots for the log observed and expected intensity values for two FMR1 chips. The intensity values are centered around their mean. **A**) Chip number 34 with an observed mean of 5,993 and an expected mean of 5,914. **B**) Chip number 32 with an observed mean of 3,054 and an expected mean of 3,058.

We used our model to predict probe intensities for seven Affymetrix re-sequencing array designs with 25 base pair long DNA probes that bind end labeled target DNA. The cwrs labeled designs come from [20]. These chips were manufactured and processed in 1999 and 2000. The seventh design, FMR1, chips come from [12]. They were manufactured and processed in 2009. The array designs have highly variable GC content, number of PCR products, and features. Mean correlations between expected and observed intensities, as well as the incorporation rates (1 - truncation rate), and the base retention rates (1 – abasic rate) for individual chips are listed in the supplementary material (Additional file 1: Table S2). For the 302 chips analyzed the mean correlations (Pearson) range from 0.881 to 0.550. For the FMR1 chip design the average correlation across all chips was 0.76. For these same chips the correlation for the log values was 0.73. Figure 4 shows the plot of the log (observed – observed mean) and log (expected – expected mean) values for chips 32 and 34 from the FMR1 design. The calculation times on a 2.4 Ghz single core CPU for K_eq_ values for each design were in the range of 30 minutes to 2 hours per PCR product. These calculations happen only once per probes set design, and do not depend in any way on the observed intensities. The running time for each individual chip was in the range of 20 minutes to 3 hours. For all chips the hybridization temperature was set to 42°C, the minimum intensity for the scanner was set to 100, and the maximum intensity was set to 65536. For all but one chip, the GOMP shape value was fitted to 7. The other chip appeared to have a GOMP value of 6.5. We ran a couple of chips using different temperature and salt concentration values and found that the temperature given by the experimental protocol plus/minus 2 degrees and a salt concentration of one molar gave the best fits. We ran each chip using 30–48 different combinations of wash and fragmentation values, and then selected the values that gave the highest correlation. There is little chance for over fitting, because the number of parameters fit (16–23) for each chip is literally 3–4 orders of magnitude lower than the number of observations for each chip (80,428 - 231,776) (Table 2). Furthermore, the maximum intensity, and minimum intensity were never fit to the data, but were rather inferred from the experimental protocol. By design, the FMR1 chip had 512 probe spots replicated in two or more places on the chip. This allowed us to estimate the correlation in observed intensity between two spots with the same probe sequence, but different positions on a single chip. The average correlation in intensity between these replicated spots is 0.906, where the average is taken over 62 different chips.

**Table 2:** Correlations

**Design**	**Number of chips**	**Number of probe spots per chip**	**Number of parameters that were fit to each chip**	**Number of PCR products**	**Mean correlation between expected and observed probe intensities**
cwrs-07	40	230640	21	9	0.765 ± 0.038
cwrs-39	40	230240	20	8	0.605 ± 0.020
cwrs-51	40	226592	23	11	0.730 ± 0.021
cwrs-53	40	230432	21	9	0.714 ± 0.016
cwrs-63	40	229280	21	9	0.666 ± 0.028
cwrs-67	40	231776	18	6	0.629 ± 0.029
fmr1	62	80408	16	4	0.762 ± 0.081

Table 2: Summary for each chip design.

For each chip design, the average correlation, the mean incorporation rates, and the mean base retention rates are listed in Tables 2, 3, and 4 respectively. We used a Kruskal-Wallis rank sum test to determine whether the incorporations rates for each nucleotide are different between chip designs. The Kruskal-Wallis chi-squared values are 175, 72, 223, and 156 for A, C, G, and T incorporations rates respectively. Each test has six degrees of freedom and a p value < 10^-12^. For A, C, G, and T base retention rates, the Kruskal-Wallis chi-squared values are 235, 154, 259, and 226 respectively. Each test has six degrees of freedom and a p value < 10^-12^. We then compared the parameters for the cwrs design chips only. For A, C, G, and T incorporation rates as well as A, C, G base retention rates each test had five degrees of freedom and a p value < 10^-12^. For T base retention rates the Kruskal-Wallis chi-square value was 16 and the p value was 0.006.

**Table 3:** Incorporation rates

**Design**	**A incorporation**	**C incorporation**	**G incorporation**	**T incorporation**
cwrs-07	0.985 ± 0.012	0.952 ± 0.017	0.916 ± 0.035	0.945 ± 0.018
cwrs-39	1.000 ± 0.000	0.950 ± 0.009	0.990 ± 0.017	0.944 ± 0.005
cwrs-51	0.999 ± 0.004	0.945 ± 0.015	0.995 ± 0.013	0.967 ± 0.008
cwrs-53	0.996 ± 0.007	0.943 ± 0.013	0.990 ± 0.015	0.973 ± 0.006
cwrs-63	0.983 ± 0.018	0.944 ± 0.010	0.943 ± 0.022	0.952 ± 0.009
cwrs-67	0.987 ± 0.012	0.931 ± 0.009	0.923 ± 0.016	0.951 ± 0.007
fmr1	0.967 ± 0.010	0.944 ± 0.015	0.908 ± 0.020	0.950 ± 0.006

Table 3: Mean incorporation rate for each nucleotide.

**Table 4:** Retention rates

**Design**	**A retention**	**C retention**	**G retention**	**T retention**
cwrs-07	0.963 ± 0.028	0.981 ± 0.013	0.865 ± 0.036	0.998 ± 0.007
cwrs-39	0.882 ± 0.005	0.930 ± 0.011	0.791 ± 0.017	1.000 ± 0.001
cwrs-51	0.904 ± 0.010	0.961 ± 0.015	0.791 ± 0.014	1.000 ± 0.000
cwrs-53	0.901 ± 0.008	0.972 ± 0.015	0.809 ± 0.015	1.000 ± 0.000
cwrs-63	0.944 ± 0.025	0.959 ± 0.013	0.866 ± 0.029	0.998 ± 0.003
cwrs-67	0.938 ± 0.013	0.956 ± 0.011	0.884 ± 0.021	0.998 ± 0.006
fmr1	0.959 ± 0.014	0.941 ± 0.027	0.953 ± 0.029	0.962 ± 0.010

Table 4: Mean retention rate for each nucleotide. (Rate abasic site = 1 – retention rate).

Intuitively, this is the primary explanation of the observed difference between forward and reverse probe intensities. If, for example, the C incorporation rates are much higher than G incorporation rates and they both have similar rates of base retention, and one probe (say the forward probe) is C rich, while the other G rich, then we will observe that the forward C rich probe will be much brighter than the complimentary reverse G rich probe. Across chip designs adenosine seems to be synthesized more efficiently than other bases. More specifically, for the FMR1 chip design adenosine synthesis is more efficient than thymidine synthesis and cytidine synthesis is more efficient than guanosine synthesis. For this chip design we looked at the ratio of forward and reverse strand intensities as a function of probe base pair composition (Additional file 2: Figure S1). For bins with more than 50 observations the observed and the expected intensity ratios followed the same trend. The deviations between the observed and the expected at the edges of the curve could be the results of small sample size and nucleotide runs that have binding affinities that deviated from those calculated using the nearest neighbor model.

**Figure 5:**
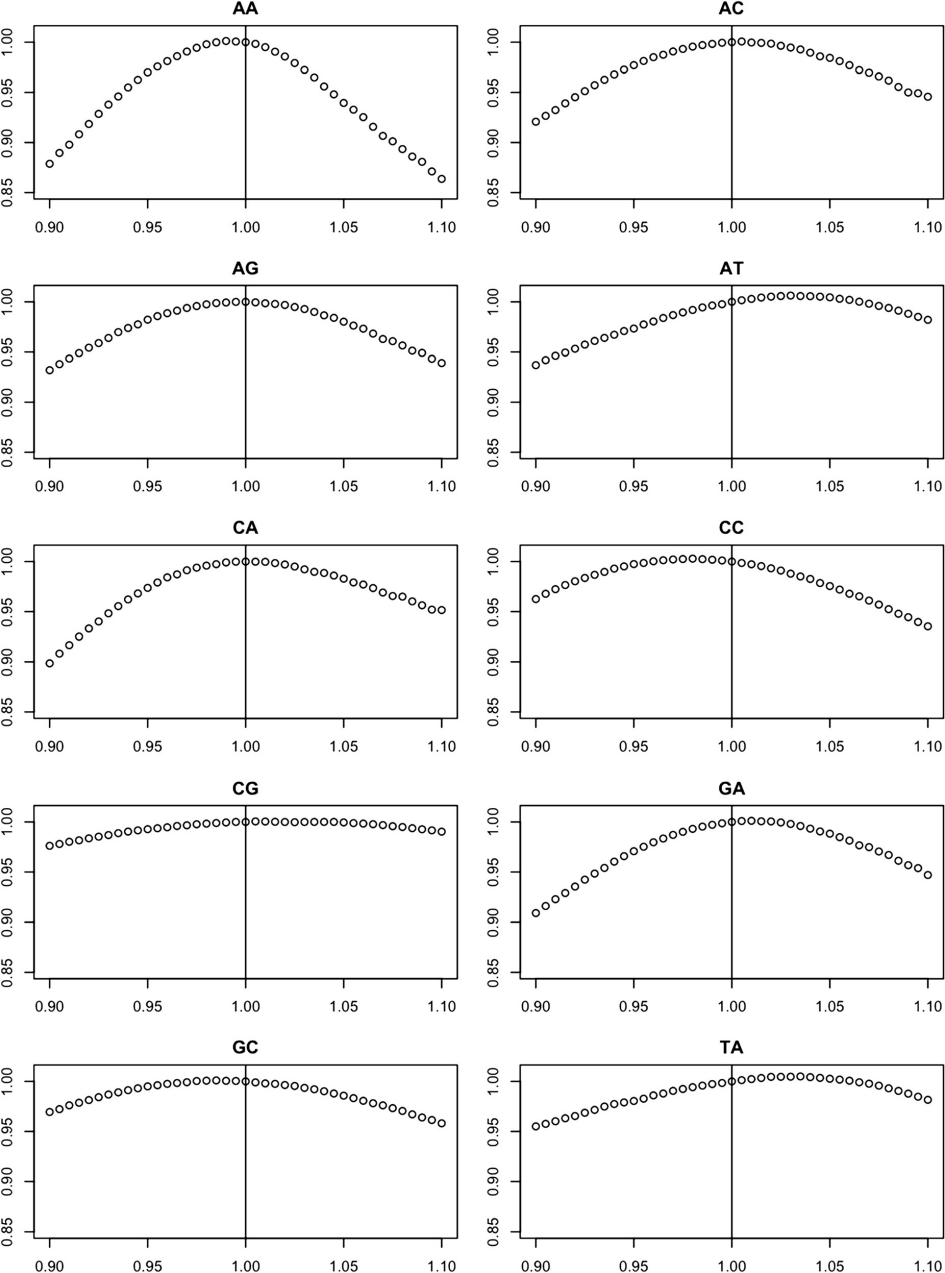
**Nearest-Neighbor Parameter Search.** Results for one dimensional search for perfect match NN values. The x-axis is the ratio of assayed ΔH value divided by the ΔH in solution value [19]. Thus, x=1 are the in solution values. The y-axis has the corresponding mean correlation divided by the mean correlation when the ΔH values are set to the in solution value [19].

To test the hypothesis that nearest-neighbor binding NN values have the same values as in solution binding [19,21-25], we ask whether or not the overall correlation between predicted and observed intensity can be improved by varying any of the binding kinetic parameters. To do so, we conducted a one-dimensional search for ΔH for all ten possible perfect match NN values, and all 51 mismatch values that have been previously estimated [21-25]. For the perfect match NN values the ΔH values that maximized the intensity correlation were essentially equal to the values estimated by Santa-Lucia *et al.*[19], and in no case was the best estimate more than 5% different from the reference values (Figure 5). On the other hand some of the ΔH values for the mismatches were modestly different from the reference values. The range of error for the mismatch values is much larger than that for the perfect matches; therefore, our estimates, which are based on thousands of observations, might represent a more accurate estimate of mismatch NN values for microarray and/or in solution binding. However, it is quite possible that these values are fundamentally confounded with manufacturing error in our model, and given the modest nature of the difference, we are not convinced that mismatch binding kinetics are substantially different from in solution binding. The linear search for optimal ΔH initiation/termination values also yielded results that were fundamentally the same as in solution kinetics. The best fit values for the mismatch NN numbers can be found in the Supplementary Materials (Additional file 1: Table S1B).

**Figure 6:**
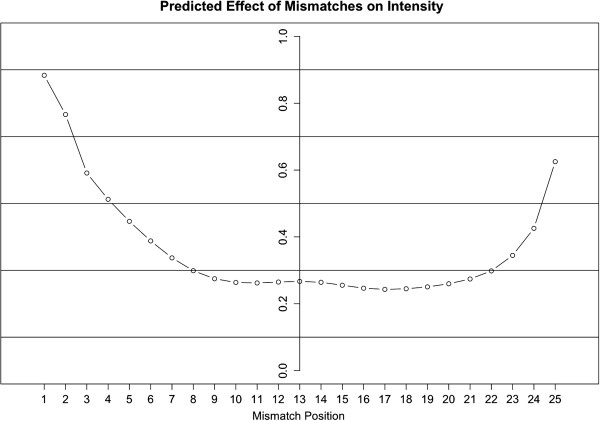
**Predicted Effect of Mismatches on Intensity.** A graph of the expected effect of each mismatch on intensity. On the x-axis is the position of the mismatch. On the y-axis is the mismatch probe intensity divided by the intensity of the corresponding probe with no mismatches, averaged across all probes.

One of the main goals of our study was to understand the effect of mismatches on probe intensity. In our model the binding affinity of a particular mismatch is independent of its position in the probe sequence. Therefore, we were interested to see if the other details in our model could explain the observation that mismatches towards the center of the probe have a larger effect on intensity compared to mismatches that are closer to the edges. Figure 6 shows the average effect of mismatches at each position on the probe. The distance of a mismatch from the edge of the probe correlates to the difference between the perfect match and mismatch intensities, with mismatches in the center having the largest effect. Mismatches towards the 3’ end have a slightly larger effect than mismatches towards the 5’ end.

## Discussion

Our model for DNA hybridization on microarrays is comprehensive. It includes parameters that are specific to the chip design and to the processing protocol. These parameters are probe length, temperature, salt concentration, wash stringency, target DNA size, and the parameters that are related to the scanners dynamic range. Furthermore, the NN values can be adjusted to accommodate DNA-RNA and RNA-RNA binding. The algorithm that calculates K_eq_ values for probe target complexes is applicable to many array designs as well as to other binding reactions in thermodynamic equilibrium. Thus, this algorithm can provide valuable information on binding affinities and cross-hybridization for a wide array of applications including probe design.

Our approach to probe intensity calculations takes into account many of the “problems” inherent to microarrays such as batch effects and probe synthesis failure. Preparation of target DNA for microarray experiments often poses many technical challenges; consequently, the concentration of DNA products often varies between experiments as does the wash strength and average fragmentation size. Furthermore, probe synthesis efficiency varies between individual chips, and different batches of arrays. Our model directly tackles this problem by fitting the concentration as well as the nucleotide synthesis parameters for each chip. It is important to note that all the chips analyzed are Affymetrix chips with 25 bp long probes; therefore, the chip synthesis aspects of our model may be somewhat specific to the company’s manufacturing process. Our model fits one parameter for the concentration of each target molecule, which limits its use for arrays that quantify transcript levels. However, the K_eq_ information from our model is applicable to any type of array.

For all the chips that were analyzed, the mean correlation between expected and observed probe intensities is 0.701, with average correlations for each chip ranging from 0.881 to 0.550. Furthermore, extreme intensity values do not dominate the correlation terms (Figure 4). For the FMR1 design chips the correlation for replicated probe spots (probe spots with the exact same probe sequence) is 0.906. Similar measurements for correlation between probe spots have been previously reported [41,42]. Thus, if we view ~90% as the maximum possible correlation between expected and observed intensity, because observed intensity varies this much between replicated spots within a single chip, our model can be seen to be doing a very good, but not quite a perfect job of predicting probe intensities.

Our model has helped us understand some puzzling observations regarding microarrays: the difference in intensity between complimentary forward and reverse probes; the larger decrease in intensity for mismatches towards the center of a probe; and batch effects.

First, assuming simple liquid phase kinetics, probe spots that target the forward DNA strand should have equal amount of bound target DNA as the complementary spot that targets the reverse DNA strand. However, the intensity of the forward and reverse strands usually have systematically different intensities, with probes targeting the forward strand of a given genomic region being brighter/darker than the set of probes that target the reverse strand of the same genomic region [20]. Under our model this observation is simply the result of probe synthesis failure or sites becoming abasic after synthesis. This claim is supported by the fact that the A,C,G, and T synthesis parameters are statistically different from each other for each of the different chip designs (Additional file 1: Table S2). The estimates for A,C,G, and T incorporation rates (Table 3) are similar to previously published estimates of synthesis failure for Affymetrix arrays [31]. Furthermore, this explanation for the difference between forward and reverse strand probes is far more parsimonious than relating this difference to G-stacks [43], given that this difference is observed in A/T rich probes that have no stretches of the same amino acid. There are two factors that we did not model, but could partially contribute to the observed difference between forward and reverse probes. For one there are base analogues that are often used to manufacture probes, this difference can easily be incorporated into our model by revising the NN numbers used for the Keq calculations. Second there could be an entropy penalty to base pairs that are closer to the array surface.

In our model, probe synthesis failure along with wash and fragmentation differences explain batch effects for microarrays. Batch effects can be subdivided into two types, those that happen during manufacturing of the chip and those that happen during the processing of the chip. In our model, the former is explained by differences in the efficiency of probe synthesis, while the latter is explained by differences in the fragmentation, washing steps, and as the rate of abasic site formation.

Another previously puzzling observation is the correlation between the distance of a mismatch from the edge of the probe and its effect on probe intensity. Mismatches towards the center have a larger effect than mismatches towards the edges [26]. Under our model this observation is expected (Figure 6), and is the result of fragmentation of the target molecule. Intuitively, there are more fragments that can bind the center of a probe, than fragments that can only bind a single edge (Figure 1). Hence, if a target molecule contains a mismatch, its effect will be proportional to its distance from the middle of the probe. In our model, simple hybridization kinetics can explain these puzzling observations without the need to assign different weights along the probe sequence nor a penalty to probes with a mismatch.

Using our model we get an average correlation of ~70% between observed and expected probe intensity. This includes data from all probe spots regardless of their quality. Even so, our model comes close (0.881) on some chips, but never achieves our theoretical maximum correlation (0.906). From the manufacturing process up until the reading of probe spots intensities, the microarray experiment has several complex steps. Our model makes several “simplistic” assumptions that allowed us to develop efficient algorithms. In doing so we made several compromises. One of these assumptions is that probes with two or more errors do not bind target DNA. This assumption should have a relatively small effect on the correlation for 25 bp long probes; however, it is expected to have a larger effect on the correlation for longer probes. In our model we use the Langmuir isotherm to calculate the fraction of bound probes and do not take into account probe surface density [16,17], non-equilibrium, and low target/probe ratio [39]. Theoretically, commercial arrays of 25 bp long probes should have reached equilibrium at the end of the hybridization step; however, equilibrium might not be achieved by the end of the washing period; therefore, our approach to modeling the wash step of the protocol is rather simplistic. Furthermore, in these arrays the target/probe ratio should be very large. When arrays deviate from this ideal scenario our model loses predictive power. It is important to note that even though the probe surface density is not directly modeled by our approach, the parameters we use to describe the scanners dynamic range can indirectly be used to adjust for microarrays with varying probe surface densities. This “adjustment” however has some limitations. The most obvious one being if increasing probe density affects mismatched sequences and matched sequences disproportionately [17].

Overall our data suggests that the Langmuir isotherm appropriately and efficiently models binding between probe and target DNA on a microarray; however, other more computationally intensive measurements for binding on arrays have been proposed [39,44]. Furthermore, when we calculate target-probe binding we do not account for the known in solution effects of dangling ends [45] and the stabilizing effect of mismatches in the last three base pairs of a sequence [21-25]. We also do not model secondary structures that can form on arrays with long probes or arrays that hybridize to targets with extensive secondary structures, for example rRNA arrays [46].

Other details of the microarray experiment that are left out of our model are bleed-through between features and regional artifacts such as air bubbles, scratches, and miscellaneous particles. There are two sources of bleed-through between features: one, the probes at the edge of a feature may have a hybrid sequence due to incorporation of nucleotides during the synthesis of the neighboring probe; two, the scanner may be detecting light from neighboring features and falsely determining its origin. If this were going on, its effect would be most noticeable in probes that would otherwise be very dark, and appears to be present (Figure 4) in our data, where a substantial fraction of probes that are predicted to have very low intensities appear to have much higher than expected intensity.

Our approach to modeling probe synthesis failure also has some limitations. First, there is the possibility that the concentration parameters are confounded with the synthesis efficiency parameters. This can be a problem when dealing with G/C or A/T rich PCR products. Second, the synthesis efficiency of a nucleotide can potentially be dependent on its position on the probe [31].

For our calculations we assume that the target DNA has the reference sequence. This assumption is never completely valid because each individual almost surely has a unique sequence that may differ slightly or even significantly from the genomic reference sequence. The impact of this assumption on the correlations for the analyzed data depends on the type of genetic variation of the samples. When the target DNA only has SNPs and/or other one base pair changes, then the genetic variation is unlikely to have a large impact on the average correlation over the entire chip; however, if the target DNA has large CNVS and/or several CNVs then these genetic variants would be expected to have a significant effect on the average correlation for the chip.

Our model is most applicable to 25 bp long arrays that are designed to detect genetic variation. With this in mind, the obvious next step is to apply our model to SNP arrays with the goal being to better determined which genetic variants are present, by incorporating our model into a variant calling algorithm.

## Competing interests

The authors declare that they have no competing interests.

## Authors’ contributions

Both authors contributed equally to all aspects of this work and the preparation of this manuscript.

## Computer programs

The computer programs described in this paper are available at https://genome.emory.edu/faculty/dcutler/.

## Supplementary Material

Additional file 1 **Table 1A.** Values for H are in J/mol. Values for S are in J/(K*mol). Click here for file

Additional file 2 **Figure 1.** For each Forward/Reverse probe pair the mean of the log (Forward/Reverse) was calculated across all 62 chips from the FMR1 design. Probes are binned by base composition with each bin corresponding to the excess of A over T nucleotides plus the excess of C over G nucleotides on the Forward strand. The numbers on the figure correspond to the number of distinct features that are observed in the bin. Observed (o) and expected means (*) are plotted for each bin. Click here for file
