# Supplementary material for: A model of binding on DNA microarrays: understanding the combined effect of probe synthesis failure, cross-hybridization, DNA fragmentation and other experimental details of affymetrix arrays
Source: BMC Genomics. 2012 Dec 27;13:737. doi: 10.1186/1471-2164-13-737 (PMC3548757; doi:10.1186/1471-2164-13-737)
Supplement: Additional file 1 — Table 1A. Values for H are in J/mol. Values for S are in J/(K*mol). [file 1471-2164-13-737-S1.pdf]

delta H	AA	AC	AG	AT	CA	CC	CG	CT	GA	GC	GT	GG	TA	TC	TG	TT
**AA**	0	0	0	9043.49	0	0	0	15407.4	0	0	0	2512.08	23613.6	31819.7	15072.5	-33076
**AC**	0	0	0	26628	0	0	0	4510	-9713.4	586.152	2093.4	-35169	0	0	0	7033.82
**AG**	0	0	0	-1172.3	-753.62	4521.74	-16747	-32657	0	0	0	-7787.5	0	0	0	6698.88
**AT**	9043.49	26628	-1172.3	-30145	0	0	0	1004.83	0	0	0	-6280.2	0	0	0	-9043.5
**CA**	0	0	-753.62	0	0	0	7954.92	0	0	0	-1758.5	0	19929.1	30647.4	-35588	8373.6
**CC**	0	0	4521.74	0	0	0	-6280.2	0	21771.4	15072.5	-33494	21771.4	0	0	-669.89	0
**CG**	0	0	-16747	0	7954.92	-6280.2	-44380	-1256	0	0	-16412	0	0	0	0	0
**CT**	15407.4	4510	-32657	1004.83	0	0	-1256	0	0	0	-7033.8	0	0	0	-20934	0
**GA**	0	-9713.4	0	0	0	21771.4	0	0	0	-2512.1	0	0	4689.22	-34332	12058	-3265.7
**GC**	0	586.152	0	0	0	15072.5	0	0	-2512.1	-41031	-20097	0	0	13481.5	0	0
**GT**	0	2093.4	0	0	-1758.5	-33494	-16412	-7033.8	0	-20097	0	0	0	13816.4	0	24283.4
**GG**	2512.08	-35169	-7787.5	-6280.2	0	21771.4	0	0	0	0	0	17165.9	0	-7368.8	0	0
**TA**	23613.6	0	0	0	19929.1	0	0	0	4689.22	0	0	0	-30145	8038.66	1004.83	2512.08
**TC**	31819.7	0	0	0	30647.4	0	0	0	-34332	13481.5	13816.4	-7368.8	8038.66	0	0	0
**TG**	15072.5	0	0	0	-35588	-669.89	0	-20934	12058	0	0	0	1004.83	0	-5861.5	0
**TT**	-33076	7033.82	6698.88	-9043.5	8373.6	0	0	0	-3265.7	0	24283.4	0	2512.08	0	0	0
**delta S**	**AA**	**AC**	**AG**	**AT**	**CA**	**CC**	**CG**	**CT**	**GA**	**GC**	**GT**	**GG**	**TA**	**TC**	**TG**	**TT**
**AA**	0	0	0	7.11756	0	0	0	19.2593	0	0	0	-9.6296	54.0097	84.5734	30.9823	-92.947
**AC**	0	0	0	61.1273	0	0	0	-18.422	-41.031	-15.91	13.3978	-93.784	0	0	0	0.83736
**AG**	0	0	0	-9.6296	-17.585	-2.5121	-55.266	-87.923	0	0	0	-39.775	0	0	0	3.76812
**AT**	7.11756	61.1273	-9.6296	-85.411	0	0	0	-25.958	0	0	0	-34.75	0	0	0	-45.217
**CA**	0	0	-17.585	0	0	0	15.4912	0	0	0	-9.6296	0	33.4944	68.6635	-95.04	2.93076
**CC**	0	0	-2.5121	0	0	0	-30.145	0	59.4526	37.2625	-83.317	56.5218	0	0	-18.841	0
**CG**	0	0	-55.266	0	15.4912	-30.145	-113.88	-25.54	0	0	-64.058	0	0	0	-48.986	0
**CT**	19.2593	-18.422	-87.923	-25.958	0	0	-25.54	0	0	0	-33.494	0	0	0	-66.151	0
**GA**	0	-41.031	0	0	0	59.4526	0	0	0	-4.1868	0	0	2.93076	-92.947	15.0725	-22.19
**GC**	0	-15.91	0	0	0	37.2625	0	0	-4.1868	-101.32	-66.151	-51.498	0	22.6087	0	0
**GT**	0	13.3978	0	0	-9.6296	-83.317	-64.058	-33.494	0	-66.151	0	0	0	43.5427	0	68.2448
**GG**	-9.6296	-93.784	-39.775	-34.75	0	56.5218	0	0	0	-51.498	0	39.7746	0	-35.169	0	0
**TA**	54.0097	0	0	0	33.4944	0	0	0	2.93076	0	0	0	-89.179	2.93076	-7.1176	-6.2802
**TC**	84.5734	0	0	0	68.6635	0	0	0	-92.947	22.6087	43.5427	-35.169	2.93076	0	0	0
**TG**	30.9823	0	0	0	-95.04	-18.841	-48.986	-66.151	15.0725	0	0	0	-7.1176	0	-25.958	0
**TT**	-92.947	0.83736	3.76812	-45.217	2.93076	0	0	0	-22.19	0	68.2448	0	-6.2802	0	0	0

delta H	A/T	G/C
**Init/Term**	9629.64	418.68
**delta S**	**A/T**	**G/C**
**Init/Term**	17.1659	-11.723

Supplementary Table 1A. Values for H are in J/mol . Values for S are in J/(K*mol).

delta H	AA	AC	AG	AT	CA	CC	CG	CT	GA	GC	GT	GG	TA	TC	TG	TT
AA	0	0	0	5024.16	0	0	0	9629.64	0	0	0	-2512.1	19678	31819.7	12560.4	-33076
AC	0	0	0	22190	0	0	0	0	-12142	-2930.8	2093.4	-35169	0	0	0	2930.76
AG	0	0	0	-2930.8	-3768.1	2512.08	-16747	-32657	0	0	0	-12979	0	0	0	4186.8
AT	5024.16	22190	-2930.8	-30145	0	0	0	-5024.2	0	0	0	-10467	0	0	0	-11304
CA	0	0	-3768.1	0	0	0	7954.92	0	0	0	-2930.8	0	14235.1	25539.5	-35588	4186.8
CC	0	0	2512.08	0	0	0	-6280.2	0	21771.4	15072.5	-33494	21771.4	0	0	-3349.4	0
CG	0	0	-16747	0	7954.92	-6280.2	-44380	-6280.2	0	0	-20515	0	0	0	-17166	0
CT	9629.64	0	-32657	-5024.2	0	0	-6280.2	0	0	0	-11723	0	0	0	-20934	0
GA	0	-12142	0	0	0	21771.4	0	0	0	-2512.1	0	0	2930.76	-34332	6698.88	-5442.8
GC	0	-2930.8	0	0	0	15072.5	0	0	-2512.1	-41031	-25121	-18422	0	9629.64	0	0
GT	0	2093.4	0	0	-2930.8	-33494	-20515	-11723	0	-25121	0	0	0	13816.4	0	24283.4
GG	-2512.1	-35169	-12979	-10467	0	21771.4	0	0	0	-18422	0	17165.9	0	-9211	0	0
TA	19678	0	0	0	14235.1	0	0	0	2930.76	0	0	0	-30145	5024.16	-418.68	837.36
TC	31819.7	0	0	0	25539.5	0	0	0	-34332	9629.64	13816.4	-9211	5024.16	0	0	0
TG	12560.4	0	0	0	-35588	-3349.4	-17166	-20934	6698.88	0	0	0	-418.68	0	-5861.5	0
TT	-33076	2930.76	4186.8	-11304	4186.8	0	0	0	-5442.8	0	24283.4	0	837.36	0	0	0
**delta S**	**AA**	**AC**	**AG**	**AT**	**CA**	**CC**	**CG**	**CT**	**GA**	**GC**	**GT**	**GG**	**TA**	**TC**	**TG**	**TT**
AA	0	0	0	7.11756	0	0	0	19.2593	0	0	0	-9.6296	54.0097	84.5734	30.9823	-92.947
AC	0	0	0	61.1273	0	0	0	-18.422	-41.031	-15.91	13.3978	-93.784	0	0	0	0.83736
AG	0	0	0	-9.6296	-17.585	-2.5121	-55.266	-87.923	0	0	0	-39.775	0	0	0	3.76812
AT	7.11756	61.1273	-9.6296	-85.411	0	0	0	-25.958	0	0	0	-34.75	0	0	0	-45.217
CA	0	0	-17.585	0	0	0	15.4912	0	0	0	-9.6296	0	33.4944	68.6635	-95.04	2.93076
CC	0	0	-2.5121	0	0	0	-30.145	0	59.4526	37.2625	-83.317	56.5218	0	0	-18.841	0
CG	0	0	-55.266	0	15.4912	-30.145	-113.88	-25.54	0	0	-64.058	0	0	0	-48.986	0
CT	19.2593	-18.422	-87.923	-25.958	0	0	-25.54	0	0	0	-33.494	0	0	0	-66.151	0
GA	0	-41.031	0	0	0	59.4526	0	0	0	-4.1868	0	0	2.93076	-92.947	15.0725	-22.19
GC	0	-15.91	0	0	0	37.2625	0	0	-4.1868	-101.32	-66.151	-51.498	0	22.6087	0	0
GT	0	13.3978	0	0	-9.6296	-83.317	-64.058	-33.494	0	-66.151	0	0	0	43.5427	0	68.2448
GG	-9.6296	-93.784	-39.775	-34.75	0	56.5218	0	0	0	-51.498	0	39.7746	0	-35.169	0	0
TA	54.0097	0	0	0	33.4944	0	0	0	2.93076	0	0	0	-89.179	2.93076	-7.1176	-6.2802
TC	84.5734	0	0	0	68.6635	0	0	0	-92.947	22.6087	43.5427	-35.169	2.93076	0	0	0
TG	30.9823	0	0	0	-95.04	-18.841	-48.986	-66.151	15.0725	0	0	0	-7.1176	0	-25.958	0
TT	-92.947	0.83736	3.76812	-45.217	2.93076	0	0	0	-22.19	0	68.2448	0	-6.2802	0	0	0

delta H	A/T	G/C
Init/Term	9629.64	418.68
**delta S**	**A/T**	**G/C**
Init/Term	17.1659	-11.723

Supplementary Table 1B

Table 2

cwrs07	*Incorporation Rate*				**Base Rate**				
**Chip**	***A***	***C***	***G***	***T***	**A**	**C**	**G**	**T**	**Correlation**
1	*0.980*	*0.963*	*0.891*	*0.934*	0.973	0.972	0.881	1.000	0.819
2	*0.968*	*0.962*	*0.879*	*0.925*	0.998	0.972	0.900	1.000	0.812
3	*0.982*	*0.959*	*0.877*	*0.919*	0.999	0.976	0.895	1.000	0.806
4	*0.973*	*0.948*	*0.878*	*0.925*	0.992	0.991	0.879	1.000	0.778
5	*0.983*	*0.953*	*0.914*	*0.966*	0.982	0.989	0.899	1.000	0.766
6	*0.981*	*0.957*	*0.909*	*0.960*	0.979	0.986	0.886	1.000	0.778
7	*0.985*	*0.959*	*0.886*	*0.942*	0.992	0.976	0.907	1.000	0.802
8	*0.988*	*0.960*	*0.914*	*0.963*	0.943	0.984	0.842	1.000	0.764
9	*0.979*	*0.967*	*0.889*	*0.940*	0.987	0.972	0.876	1.000	0.797
10	*0.970*	*0.965*	*0.879*	*0.935*	1.000	0.970	0.884	1.000	0.804
11	*1.000*	*0.969*	*0.955*	*0.957*	0.932	0.983	0.863	0.987	0.764
12	*0.976*	*0.959*	*0.869*	*0.918*	1.000	0.962	0.915	1.000	0.817
13	*0.955*	*0.974*	*0.965*	*0.947*	0.984	0.984	0.847	0.987	0.769
14	*1.000*	*0.963*	*0.963*	*0.967*	0.925	0.979	0.826	1.000	0.758
15	*0.991*	*0.947*	*0.953*	*0.965*	0.930	1.000	0.829	1.000	0.732
16	*0.999*	*0.947*	*0.936*	*0.959*	0.926	1.000	0.833	1.000	0.773
17	*0.973*	*0.962*	*0.899*	*0.935*	0.982	0.973	0.876	1.000	0.773
18	*0.979*	*0.962*	*0.912*	*0.949*	0.954	0.978	0.848	1.000	0.764
19	*0.995*	*0.971*	*0.976*	*0.966*	0.935	0.953	0.820	1.000	0.722
20	*0.990*	*0.962*	*0.964*	*0.964*	0.937	0.972	0.828	1.000	0.730
21	*0.993*	*0.943*	*0.952*	*0.966*	0.939	0.991	0.832	1.000	0.735
22	*1.000*	*0.949*	*0.931*	*0.950*	0.928	0.977	0.851	1.000	0.727
23	*1.000*	*0.943*	*0.851*	*0.925*	0.997	0.976	0.962	0.964	0.828
24	*0.973*	*0.965*	*0.906*	*0.932*	0.999	0.967	0.900	1.000	0.786
25	*0.992*	*0.933*	*0.906*	*0.949*	0.943	1.000	0.852	1.000	0.730
26	*0.982*	*0.958*	*0.888*	*0.922*	0.977	0.963	0.877	1.000	0.785
27	*0.981*	*0.940*	*0.943*	*0.971*	0.959	0.997	0.848	1.000	0.742
28	*0.989*	*0.939*	*0.940*	*0.966*	0.945	1.000	0.844	1.000	0.720
29	*1.000*	*0.949*	*0.971*	*0.953*	0.918	0.980	0.809	1.000	0.743
30	*0.986*	*0.959*	*0.968*	*0.964*	0.946	0.968	0.828	1.000	0.721
31	*1.000*	*0.933*	*0.958*	*0.962*	0.926	0.999	0.825	1.000	0.725
32	*0.969*	*0.961*	*0.890*	*0.934*	1.000	0.970	0.886	1.000	0.785
33	*0.996*	*0.925*	*0.930*	*0.951*	0.932	0.996	0.829	1.000	0.678
34	*0.991*	*0.955*	*0.852*	*0.905*	1.000	0.960	0.951	0.988	0.824
35	*0.973*	*0.910*	*0.928*	*0.933*	0.956	0.992	0.824	1.000	0.705
36	*0.991*	*0.971*	*0.906*	*0.941*	0.943	0.968	0.851	1.000	0.788
37	*0.982*	*0.962*	*0.906*	*0.934*	0.983	0.983	0.905	1.000	0.775
38	*0.980*	*0.965*	*0.915*	*0.948*	0.967	0.982	0.883	1.000	0.825
39	*1.000*	*0.943*	*0.923*	*0.951*	0.928	0.993	0.840	1.000	0.764
40	*0.957*	*0.888*	*0.873*	*0.912*	0.963	0.999	0.858	0.982	0.697
**Mean**	***0.985***	***0.952***	***0.916***	***0.945***	**0.963**	**0.981**	**0.865**	**0.998**	**0.765**
**Std. Dev.**	***0.012***	***0.017***	***0.035***	***0.018***	**0.028**	**0.013**	**0.036**	**0.007**	**0.038**
**Max.**	***1.000***	***0.974***	***0.976***	***0.971***	**1.000**	**1.000**	**0.962**	**1.000**	**0.828**
**Min.**	***0.955***	***0.888***	***0.851***	***0.905***	**0.918**	**0.953**	**0.809**	**0.964**	**0.678**

cwrs39	*Incorporation Rate*				**Base Rate**				
**Chip**	***A***	***C***	***G***	***T***	**A**	**C**	**G**	**T**	**Correlation**
1	*1.000*	*0.954*	*1.000*	*0.949*	0.883	0.936	0.794	1.000	0.620
2	*1.000*	*0.944*	*0.999*	*0.943*	0.882	0.937	0.780	1.000	0.608
3	*1.000*	*0.960*	*0.975*	*0.946*	0.889	0.917	0.810	0.999	0.550
4	*1.000*	*0.953*	*0.975*	*0.948*	0.886	0.928	0.804	1.000	0.603
5	*1.000*	*0.949*	*0.997*	*0.949*	0.881	0.937	0.784	1.000	0.612
6	*1.000*	*0.956*	*0.998*	*0.941*	0.880	0.921	0.778	1.000	0.604
7	*1.000*	*0.950*	*1.000*	*0.939*	0.874	0.925	0.774	1.000	0.615
8	*1.000*	*0.946*	*0.986*	*0.946*	0.886	0.935	0.793	1.000	0.603
9	*1.000*	*0.945*	*1.000*	*0.947*	0.884	0.936	0.781	1.000	0.617
10	*1.000*	*0.955*	*1.000*	*0.939*	0.882	0.921	0.775	1.000	0.595
11	*1.000*	*0.953*	*0.997*	*0.948*	0.883	0.930	0.784	1.000	0.616
12	*1.000*	*0.968*	*0.981*	*0.956*	0.891	0.917	0.820	1.000	0.622
13	*1.000*	*0.948*	*0.997*	*0.944*	0.887	0.929	0.786	1.000	0.585
14	*1.000*	*0.943*	*1.000*	*0.949*	0.880	0.937	0.779	1.000	0.618
15	*1.000*	*0.948*	*0.994*	*0.946*	0.886	0.931	0.787	1.000	0.595
16	*1.000*	*0.951*	*0.999*	*0.948*	0.884	0.928	0.784	1.000	0.595
17	*1.000*	*0.949*	*1.000*	*0.950*	0.883	0.932	0.785	0.999	0.606
18	*1.000*	*0.955*	*0.998*	*0.951*	0.881	0.928	0.787	0.999	0.610
19	*1.000*	*0.940*	*0.990*	*0.942*	0.883	0.935	0.786	1.000	0.611
20	*1.000*	*0.943*	*0.996*	*0.949*	0.883	0.940	0.779	1.000	0.635
21	*1.000*	*0.952*	*0.994*	*0.947*	0.884	0.928	0.790	1.000	0.589
22	*0.998*	*0.954*	*0.972*	*0.939*	0.899	0.927	0.810	1.000	0.672
23	*1.000*	*0.959*	*0.996*	*0.946*	0.887	0.922	0.789	0.997	0.599
24	*1.000*	*0.926*	*0.928*	*0.943*	0.882	0.955	0.849	1.000	0.594
25	*1.000*	*0.953*	*1.000*	*0.946*	0.881	0.929	0.782	1.000	0.610
26	*1.000*	*0.951*	*1.000*	*0.946*	0.873	0.929	0.782	1.000	0.625
27	*1.000*	*0.951*	*0.990*	*0.942*	0.878	0.926	0.784	1.000	0.611
28	*1.000*	*0.958*	*1.000*	*0.934*	0.871	0.914	0.776	1.000	0.592
29	*1.000*	*0.939*	*0.933*	*0.937*	0.880	0.939	0.839	1.000	0.593
30	*1.000*	*0.937*	*0.953*	*0.936*	0.881	0.935	0.819	1.000	0.569
31	*0.999*	*0.951*	*0.997*	*0.934*	0.876	0.917	0.780	1.000	0.586
32	*1.000*	*0.954*	*1.000*	*0.937*	0.874	0.919	0.775	1.000	0.603
33	*1.000*	*0.943*	*0.982*	*0.937*	0.882	0.932	0.794	1.000	0.582
34	*1.000*	*0.919*	*0.983*	*0.948*	0.887	0.970	0.801	1.000	0.605
35	*1.000*	*0.962*	*0.997*	*0.931*	0.873	0.899	0.783	0.995	0.578
36	*1.000*	*0.955*	*0.994*	*0.947*	0.892	0.930	0.791	1.000	0.618
37	*1.000*	*0.948*	*0.998*	*0.948*	0.885	0.935	0.782	1.000	0.618
38	*1.000*	*0.958*	*0.994*	*0.949*	0.882	0.923	0.793	0.999	0.612
39	*1.000*	*0.949*	*0.996*	*0.946*	0.881	0.932	0.781	1.000	0.617
40	*1.000*	*0.950*	*0.997*	*0.945*	0.880	0.934	0.780	1.000	0.613
**Mean**	***1.000***	***0.950***	***0.990***	***0.944***	**0.882**	**0.930**	**0.791**	**1.000**	**0.605**
**Std. Dev.**	***0.000***	***0.009***	***0.017***	***0.005***	**0.005**	**0.011**	**0.017**	**0.001**	**0.020**
**Max.**	***1.000***	***0.968***	***1.000***	***0.956***	**0.899**	**0.970**	**0.849**	**1.000**	**0.672**
**Min.**	***0.998***	***0.919***	***0.928***	***0.931***	**0.871**	**0.899**	**0.774**	**0.995**	**0.550**

cwrs51	*Incorporation Rate*				**Base Rate**				
**Chip**	***A***	***C***	***G***	***T***	**A**	**C**	**G**	**T**	**Correlation**
1	*0.991*	*0.968*	*0.988*	*0.973*	0.919	0.938	0.795	1.000	0.743
2	*1.000*	*0.932*	*1.000*	*0.952*	0.889	0.959	0.776	1.000	0.718
3	*0.984*	*0.967*	*0.978*	*0.974*	0.930	0.939	0.804	1.000	0.732
4	*1.000*	*0.943*	*0.988*	*0.973*	0.913	0.966	0.793	1.000	0.736
5	*1.000*	*0.934*	*0.990*	*0.968*	0.899	0.978	0.789	1.000	0.739
6	*1.000*	*0.933*	*1.000*	*0.963*	0.900	0.971	0.783	1.000	0.718
7	*1.000*	*0.948*	*1.000*	*0.971*	0.904	0.957	0.786	1.000	0.740
8	*1.000*	*0.953*	*0.998*	*0.963*	0.891	0.956	0.779	1.000	0.749
9	*1.000*	*0.931*	*1.000*	*0.964*	0.907	0.966	0.784	1.000	0.691
10	*1.000*	*0.936*	*0.996*	*0.966*	0.899	0.976	0.785	1.000	0.732
11	*1.000*	*0.936*	*0.996*	*0.965*	0.896	0.974	0.785	1.000	0.734
12	*1.000*	*0.960*	*1.000*	*0.974*	0.913	0.949	0.791	1.000	0.733
13	*1.000*	*0.947*	*0.924*	*0.978*	0.910	0.965	0.867	1.000	0.724
14	*1.000*	*0.931*	*1.000*	*0.966*	0.904	0.976	0.786	1.000	0.720
15	*1.000*	*0.940*	*0.982*	*0.971*	0.905	0.971	0.809	1.000	0.738
16	*1.000*	*0.969*	*0.999*	*0.979*	0.909	0.939	0.791	1.000	0.766
17	*1.000*	*0.934*	*1.000*	*0.946*	0.881	0.961	0.780	1.000	0.718
18	*0.985*	*0.959*	*1.000*	*0.970*	0.919	0.948	0.788	1.000	0.734
19	*1.000*	*0.939*	*0.993*	*0.967*	0.903	0.969	0.794	1.000	0.731
20	*1.000*	*0.941*	*0.994*	*0.953*	0.903	0.951	0.792	1.000	0.720
21	*1.000*	*0.928*	*1.000*	*0.965*	0.898	0.973	0.786	1.000	0.712
22	*1.000*	*0.944*	*1.000*	*0.966*	0.899	0.959	0.784	1.000	0.724
23	*1.000*	*0.949*	*1.000*	*0.978*	0.905	0.968	0.788	1.000	0.749
24	*0.991*	*0.965*	*0.979*	*0.976*	0.926	0.935	0.810	1.000	0.697
25	*1.000*	*0.920*	*1.000*	*0.960*	0.897	0.979	0.786	1.000	0.694
26	*1.000*	*0.956*	*1.000*	*0.970*	0.904	0.955	0.793	1.000	0.741
27	*0.995*	*0.936*	*1.000*	*0.967*	0.905	0.975	0.792	1.000	0.722
28	*0.999*	*0.957*	*1.000*	*0.968*	0.904	0.952	0.792	1.000	0.724
29	*1.000*	*0.965*	*1.000*	*0.957*	0.899	0.941	0.796	1.000	0.712
30	*1.000*	*0.929*	*1.000*	*0.966*	0.907	0.979	0.791	1.000	0.721
31	*1.000*	*0.932*	*1.000*	*0.971*	0.906	0.976	0.790	1.000	0.727
32	*1.000*	*0.919*	*1.000*	*0.957*	0.892	0.970	0.777	1.000	0.700
33	*1.000*	*0.929*	*1.000*	*0.955*	0.903	0.966	0.783	1.000	0.705
34	*1.000*	*0.956*	*0.997*	*0.979*	0.910	0.964	0.793	1.000	0.755
35	*1.000*	*0.984*	*1.000*	*0.977*	0.919	0.913	0.795	1.000	0.725
36	*1.000*	*0.953*	*1.000*	*0.974*	0.903	0.958	0.784	1.000	0.749
37	*1.000*	*0.938*	*1.000*	*0.949*	0.884	0.958	0.776	1.000	0.727
38	*1.000*	*0.953*	*0.995*	*0.970*	0.895	0.965	0.787	1.000	0.767
39	*1.000*	*0.949*	*1.000*	*0.978*	0.908	0.968	0.784	1.000	0.796
40	*1.000*	*0.933*	*1.000*	*0.970*	0.907	0.974	0.784	1.000	0.730
**Mean**	***0.999***	***0.945***	***0.995***	***0.967***	**0.904**	**0.961**	**0.791**	**1.000**	**0.730**
**Std. Dev.**	***0.004***	***0.015***	***0.013***	***0.008***	**0.010**	**0.015**	**0.014**	**0.000**	**0.021**
**Max.**	***1.000***	***0.984***	***1.000***	***0.979***	**0.930**	**0.979**	**0.867**	**1.000**	**0.796**
**Min.**	***0.984***	***0.919***	***0.924***	***0.946***	**0.881**	**0.913**	**0.776**	**1.000**	**0.691**

cwrs53	*Incorporation Rate*				Base Rate				
**Chip**	***A***	***C***	***G***	***T***	**A**	**C**	**G**	**T**	**Correlation**
1	1.000	0.954	1.000	0.971	0.897	0.959	0.800	1.000	0.704
2	0.984	0.948	1.000	0.975	0.914	0.972	0.808	1.000	0.689
3	1.000	0.954	0.990	0.964	0.902	0.960	0.811	1.000	0.729
4	1.000	0.968	1.000	0.980	0.905	0.937	0.797	1.000	0.721
5	1.000	0.930	1.000	0.974	0.895	0.988	0.793	1.000	0.705
6	1.000	0.936	0.989	0.977	0.897	0.978	0.806	1.000	0.727
7	1.000	0.928	0.990	0.970	0.902	0.991	0.806	1.000	0.704
8	1.000	0.964	1.000	0.967	0.886	0.940	0.793	1.000	0.705
9	0.983	0.962	1.000	0.975	0.909	0.952	0.798	1.000	0.701
10	1.000	0.940	0.983	0.974	0.892	0.977	0.811	1.000	0.713
11	1.000	0.941	0.974	0.974	0.893	0.976	0.821	1.000	0.681
12	1.000	0.957	1.000	0.977	0.905	0.958	0.804	1.000	0.699
13	1.000	0.935	0.973	0.968	0.900	0.976	0.825	1.000	0.742
14	0.968	0.947	1.000	0.977	0.927	0.974	0.803	1.000	0.733
15	0.998	0.933	1.000	0.976	0.893	0.979	0.803	1.000	0.744
16	1.000	0.947	1.000	0.980	0.901	0.973	0.804	1.000	0.718
17	1.000	0.925	0.980	0.964	0.905	0.997	0.823	1.000	0.738
18	1.000	0.934	0.995	0.958	0.896	0.977	0.804	1.000	0.719
19	0.989	0.945	1.000	0.971	0.905	0.973	0.797	1.000	0.725
20	1.000	0.933	0.974	0.974	0.889	0.991	0.808	0.999	0.732
21	0.997	0.933	0.947	0.967	0.911	0.977	0.851	1.000	0.733
22	1.000	0.967	0.997	0.969	0.909	0.944	0.809	1.000	0.727
23	1.000	0.935	0.993	0.977	0.906	0.988	0.811	1.000	0.697
24	0.979	0.943	1.000	0.975	0.913	0.974	0.797	1.000	0.696
25	1.000	0.935	0.955	0.973	0.901	0.974	0.838	1.000	0.718
26	0.989	0.955	1.000	0.979	0.898	0.964	0.798	1.000	0.720
27	1.000	0.933	0.998	0.971	0.899	0.977	0.798	1.000	0.686
28	0.992	0.948	1.000	0.975	0.892	0.968	0.796	1.000	0.723
29	1.000	0.941	1.000	0.971	0.898	0.970	0.797	1.000	0.728
30	1.000	0.941	1.000	0.975	0.899	0.964	0.797	1.000	0.728
31	1.000	0.935	1.000	0.970	0.894	0.981	0.799	1.000	0.691
32	1.000	0.939	0.995	0.971	0.900	0.971	0.805	1.000	0.695
33	1.000	0.916	0.958	0.954	0.912	0.992	0.841	1.000	0.714
34	0.991	0.945	0.964	0.981	0.904	0.979	0.838	1.000	0.695
35	1.000	0.928	0.989	0.975	0.896	0.985	0.813	1.000	0.726
36	0.992	0.936	0.968	0.979	0.905	0.990	0.835	1.000	0.699
37	0.994	0.975	1.000	0.976	0.911	0.933	0.797	1.000	0.716
38	0.997	0.941	0.983	0.976	0.897	0.983	0.816	1.000	0.704
39	1.000	0.957	1.000	0.977	0.896	0.961	0.798	1.000	0.718
40	0.988	0.941	1.000	0.975	0.907	0.981	0.802	1.000	0.711
**Mean**	***0.996***	***0.943***	***0.990***	***0.973***	**0.901**	***0.972***	***0.809***	***1.000***	***0.714***
**Std. Dev.**	***0.007***	***0.013***	***0.015***	***0.006***	**0.008**	***0.015***	***0.015***	***0.000***	***0.016***
**Max.**	***1.000***	***0.975***	***1.000***	***0.981***	**0.927**	***0.997***	***0.851***	***1.000***	***0.744***
**Min.**	***0.968***	***0.916***	***0.947***	***0.954***	**0.886**	***0.933***	***0.793***	***0.999***	***0.681***

cwrs63	*Incorporation Rate*				**Base Rate**				
**Chip**	***A***	***C***	***G***	***T***	**A**	**C**	**G**	**T**	**Correlation**
1	*0.994*	*0.965*	*0.948*	*0.942*	0.948	0.933	0.870	1.000	0.646
2	*1.000*	*0.932*	*0.927*	*0.963*	0.910	0.972	0.884	1.000	0.614
3	*1.000*	*0.934*	*0.934*	*0.960*	0.920	0.973	0.867	1.000	0.644
4	*0.988*	*0.949*	*0.959*	*0.950*	0.936	0.949	0.843	1.000	0.655
5	*1.000*	*0.947*	*0.953*	*0.956*	0.924	0.949	0.852	0.998	0.667
6	*1.000*	*0.938*	*0.924*	*0.961*	0.906	0.962	0.875	1.000	0.608
7	*0.945*	*0.938*	*0.917*	*0.968*	0.987	0.978	0.907	1.000	0.618
8	*0.961*	*0.939*	*0.949*	*0.957*	0.959	0.968	0.856	1.000	0.667
9	*1.000*	*0.948*	*0.953*	*0.949*	0.927	0.950	0.851	1.000	0.679
10	*0.977*	*0.949*	*0.951*	*0.952*	0.943	0.962	0.856	1.000	0.680
11	*0.982*	*0.943*	*0.972*	*0.955*	0.925	0.962	0.821	1.000	0.630
12	*0.999*	*0.969*	*0.933*	*0.949*	0.939	0.930	0.885	1.000	0.692
13	*0.953*	*0.932*	*0.898*	*0.952*	0.998	0.983	0.927	1.000	0.621
14	*1.000*	*0.960*	*0.991*	*0.950*	0.928	0.941	0.818	0.997	0.675
15	*0.978*	*0.939*	*0.993*	*0.946*	0.936	0.967	0.806	1.000	0.678
16	*0.976*	*0.939*	*0.962*	*0.942*	0.955	0.963	0.847	1.000	0.709
17	*1.000*	*0.944*	*0.958*	*0.945*	0.931	0.955	0.844	0.995	0.683
18	*0.979*	*0.939*	*0.943*	*0.939*	0.956	0.955	0.865	1.000	0.689
19	*1.000*	*0.943*	*0.946*	*0.951*	0.925	0.956	0.858	0.999	0.674
20	*0.970*	*0.941*	*0.958*	*0.953*	0.950	0.962	0.844	1.000	0.687
21	*0.965*	*0.924*	*0.908*	*0.938*	0.984	0.964	0.916	0.984	0.692
22	*1.000*	*0.976*	*0.966*	*0.960*	0.930	0.922	0.845	1.000	0.651
23	*0.990*	*0.945*	*0.921*	*0.944*	0.959	0.952	0.906	0.992	0.718
24	*0.956*	*0.943*	*0.923*	*0.960*	0.980	0.973	0.896	1.000	0.652
25	*1.000*	*0.949*	*0.974*	*0.968*	0.908	0.951	0.828	0.993	0.660
26	*1.000*	*0.937*	*0.954*	*0.958*	0.908	0.960	0.847	1.000	0.626
27	*0.993*	*0.943*	*0.931*	*0.940*	0.943	0.945	0.874	0.996	0.697
28	*1.000*	*0.943*	*0.929*	*0.967*	0.901	0.961	0.870	1.000	0.660
29	*0.942*	*0.949*	*0.918*	*0.971*	0.993	0.967	0.905	1.000	0.637
30	*0.995*	*0.942*	*0.932*	*0.945*	0.942	0.958	0.879	0.997	0.638
31	*0.975*	*0.941*	*0.977*	*0.951*	0.936	0.963	0.818	1.000	0.657
32	*1.000*	*0.950*	*0.943*	*0.946*	0.924	0.949	0.857	1.000	0.677
33	*0.964*	*0.940*	*0.963*	*0.955*	0.957	0.965	0.841	1.000	0.673
34	*0.970*	*0.941*	*0.921*	*0.945*	0.968	0.965	0.896	1.000	0.676
35	*0.965*	*0.938*	*0.918*	*0.953*	0.952	0.963	0.884	1.000	0.687
36	*0.965*	*0.938*	*0.933*	*0.948*	0.970	0.966	0.876	1.000	0.661
37	*0.987*	*0.948*	*0.931*	*0.948*	0.960	0.950	0.888	0.989	0.710
38	*1.000*	*0.941*	*0.952*	*0.943*	0.927	0.959	0.847	1.000	0.667
39	*0.981*	*0.935*	*0.921*	*0.939*	0.966	0.973	0.907	1.000	0.709
40	*0.964*	*0.940*	*0.917*	*0.949*	0.969	0.969	0.898	1.000	0.656
**Mean**	***0.983***	***0.944***	***0.943***	***0.952***	**0.944**	**0.959**	**0.866**	**0.998**	**0.666**
**Std. Dev.**	***0.018***	***0.010***	***0.022***	***0.009***	**0.025**	**0.013**	**0.029**	**0.003**	**0.028**
**Max.**	***1.000***	***0.976***	***0.993***	***0.971***	**0.998**	**0.983**	**0.927**	**1.000**	**0.718**
**Min.**	***0.942***	***0.924***	***0.898***	***0.938***	**0.901**	**0.922**	**0.806**	**0.984**	**0.608**

cwrs67	*Incorporation Rate*				Base Rate				
Chip	*A*	*C*	*G*	*T*	A	C	G	T	Correlation
1	*0.987*	*0.940*	*0.924*	*0.945*	0.947	0.939	0.892	1.000	0.625
2	*0.991*	*0.960*	*0.961*	*0.957*	0.924	0.929	0.839	1.000	0.596
3	*1.000*	*0.932*	*0.938*	*0.956*	0.925	0.962	0.872	0.994	0.641
4	*1.000*	*0.953*	*0.957*	*0.957*	0.923	0.935	0.846	1.000	0.607
5	*0.988*	*0.923*	*0.919*	*0.943*	0.943	0.964	0.892	1.000	0.596
6	*1.000*	*0.934*	*0.908*	*0.955*	0.923	0.960	0.902	1.000	0.585
7	*1.000*	*0.939*	*0.927*	*0.956*	0.923	0.952	0.881	1.000	0.621
8	*0.995*	*0.929*	*0.919*	*0.946*	0.934	0.962	0.895	1.000	0.693
9	*1.000*	*0.928*	*0.918*	*0.946*	0.925	0.960	0.885	1.000	0.659
10	*1.000*	*0.941*	*0.922*	*0.966*	0.912	0.954	0.884	1.000	0.647
11	*0.998*	*0.917*	*0.906*	*0.945*	0.928	0.966	0.903	0.997	0.639
12	*0.989*	*0.942*	*0.921*	*0.947*	0.954	0.936	0.905	0.990	0.627
13	*0.980*	*0.935*	*0.922*	*0.951*	0.939	0.954	0.875	1.000	0.607
14	*0.974*	*0.916*	*0.887*	*0.941*	0.967	0.965	0.933	0.991	0.611
15	*0.993*	*0.921*	*0.901*	*0.941*	0.940	0.964	0.910	1.000	0.673
16	*0.980*	*0.935*	*0.945*	*0.956*	0.935	0.962	0.856	1.000	0.607
17	*1.000*	*0.933*	*0.926*	*0.952*	0.923	0.951	0.880	1.000	0.641
18	*0.967*	*0.930*	*0.923*	*0.959*	0.949	0.963	0.877	1.000	0.598
19	*1.000*	*0.928*	*0.935*	*0.953*	0.923	0.960	0.872	1.000	0.641
20	*1.000*	*0.931*	*0.920*	*0.950*	0.927	0.953	0.890	0.998	0.625
21	*0.990*	*0.927*	*0.907*	*0.945*	0.937	0.957	0.899	1.000	0.629
22	*0.984*	*0.923*	*0.936*	*0.950*	0.934	0.963	0.864	1.000	0.600
23	*0.985*	*0.918*	*0.909*	*0.943*	0.957	0.968	0.914	1.000	0.669
24	*0.989*	*0.916*	*0.899*	*0.940*	0.940	0.968	0.910	1.000	0.630
25	*0.997*	*0.927*	*0.932*	*0.949*	0.925	0.960	0.874	1.000	0.647
26	*1.000*	*0.931*	*0.919*	*0.948*	0.926	0.958	0.890	1.000	0.610
27	*0.965*	*0.932*	*0.955*	*0.963*	0.944	0.960	0.846	1.000	0.569
28	*0.970*	*0.930*	*0.928*	*0.955*	0.948	0.960	0.875	1.000	0.622
29	*0.983*	*0.925*	*0.914*	*0.946*	0.944	0.963	0.887	1.000	0.600
30	*0.975*	*0.929*	*0.916*	*0.952*	0.950	0.962	0.887	1.000	0.578
31	*0.981*	*0.933*	*0.915*	*0.941*	0.957	0.946	0.902	1.000	0.624
32	*0.991*	*0.919*	*0.903*	*0.939*	0.943	0.962	0.910	1.000	0.622
33	*0.967*	*0.933*	*0.920*	*0.962*	0.943	0.957	0.881	1.000	0.642
34	*0.984*	*0.935*	*0.914*	*0.943*	0.959	0.942	0.902	1.000	0.684
35	*0.987*	*0.930*	*0.927*	*0.951*	0.927	0.958	0.865	1.000	0.663
36	*0.982*	*0.937*	*0.915*	*0.951*	0.960	0.926	0.903	0.963	0.672
37	*0.963*	*0.937*	*0.943*	*0.961*	0.946	0.961	0.854	1.000	0.618
38	*0.963*	*0.936*	*0.942*	*0.962*	0.950	0.961	0.854	1.000	0.647
39	*1.000*	*0.934*	*0.937*	*0.954*	0.922	0.959	0.868	1.000	0.668
40	*0.973*	*0.928*	*0.920*	*0.954*	0.947	0.965	0.880	1.000	0.637
**Mean**	***0.987***	***0.931***	***0.923***	***0.951***	**0.938**	**0.956**	**0.884**	**0.998**	**0.629**
**Std. Dev.**	***0.012***	***0.009***	***0.016***	***0.007***	**0.013**	**0.011**	**0.021**	**0.006**	**0.029**
**Max.**	***1.000***	***0.960***	***0.961***	***0.966***	**0.967**	**0.968**	**0.933**	**1.000**	**0.693**
**Min.**	***0.963***	***0.916***	***0.887***	***0.939***	**0.912**	**0.926**	**0.839**	**0.963**	**0.569**

fmr1	*Incorporation Rate*				Base Rate				
**Chip**	***A***	***C***	***G***	***T***	**A**	**C**	**G**	**T**	**Correlation**
1	*0.955*	*0.933*	*0.887*	*0.938*	0.972	0.975	0.983	0.971	0.810
2	*0.963*	*0.944*	*0.885*	*0.943*	0.966	0.961	0.995	0.979	0.881
3	*0.967*	*0.955*	*0.920*	*0.955*	0.971	0.945	0.956	0.958	0.848
4	*0.970*	*0.939*	*0.908*	*0.949*	0.962	0.965	0.969	0.965	0.850
5	*0.972*	*0.936*	*0.875*	*0.953*	0.960	0.966	1.000	0.946	0.850
6	*0.955*	*0.930*	*0.884*	*0.944*	0.970	0.971	0.980	0.960	0.836
7	*0.956*	*0.933*	*0.895*	*0.943*	0.978	0.968	0.969	0.967	0.826
8	*0.960*	*0.943*	*0.898*	*0.949*	0.973	0.943	0.955	0.954	0.873
9	*0.962*	*0.939*	*0.891*	*0.948*	0.971	0.965	0.986	0.956	0.839
10	*0.964*	*0.943*	*0.896*	*0.950*	0.971	0.959	0.985	0.967	0.868
11	*0.965*	*0.948*	*0.907*	*0.951*	0.968	0.963	0.970	0.962	0.852
12	*0.989*	*0.972*	*0.934*	*0.959*	0.948	0.909	0.925	0.948	0.834
13	*0.979*	*0.968*	*0.912*	*0.961*	0.960	0.908	0.941	0.936	0.836
14	*0.949*	*0.928*	*0.878*	*0.941*	0.970	0.980	0.990	0.957	0.824
15	*0.985*	*0.928*	*0.926*	*0.956*	0.920	0.951	0.913	0.971	0.656
16	*0.957*	*0.921*	*0.882*	*0.940*	0.971	0.972	0.989	0.967	0.734
17	*0.969*	*0.949*	*0.916*	*0.948*	0.957	0.959	0.952	0.961	0.827
18	*0.971*	*0.941*	*0.909*	*0.953*	0.942	0.945	0.950	0.971	0.677
19	*0.962*	*0.942*	*0.891*	*0.948*	0.971	0.968	0.987	0.962	0.845
20	*0.974*	*0.938*	*0.906*	*0.948*	0.943	0.941	0.930	0.972	0.746
21	*0.972*	*0.952*	*0.916*	*0.954*	0.952	0.923	0.943	0.966	0.679
22	*0.994*	*0.999*	*0.981*	*0.961*	0.942	0.873	0.881	0.955	0.701
23	*0.961*	*0.941*	*0.899*	*0.943*	0.956	0.916	0.946	0.958	0.642
24	*0.972*	*0.945*	*0.923*	*0.950*	0.958	0.929	0.929	0.968	0.716
25	*0.974*	*0.937*	*0.921*	*0.949*	0.947	0.942	0.926	0.972	0.714
26	*0.957*	*0.932*	*0.879*	*0.943*	0.966	0.931	0.980	0.964	0.654
27	*0.975*	*0.955*	*0.920*	*0.960*	0.962	0.903	0.932	0.951	0.769
28	*0.964*	*0.937*	*0.911*	*0.946*	0.955	0.931	0.939	0.965	0.662
29	*0.974*	*0.960*	*0.930*	*0.955*	0.952	0.905	0.936	0.957	0.639
30	*0.972*	*0.969*	*0.933*	*0.953*	0.951	0.906	0.924	0.969	0.664
31	*0.960*	*0.941*	*0.905*	*0.945*	0.962	0.919	0.948	0.955	0.701
32	*0.960*	*0.937*	*0.877*	*0.942*	0.958	0.942	0.981	0.949	0.756
33	*0.968*	*0.943*	*0.907*	*0.951*	0.971	0.961	0.975	0.958	0.818
34	*0.968*	*0.941*	*0.908*	*0.955*	0.971	0.951	0.976	0.946	0.855
35	*0.972*	*0.952*	*0.927*	*0.950*	0.959	0.917	0.929	0.968	0.726
36	*0.974*	*0.950*	*0.923*	*0.954*	0.952	0.923	0.931	0.962	0.711
37	*0.960*	*0.925*	*0.911*	*0.939*	0.957	0.967	0.939	0.989	0.697
38	*0.971*	*0.961*	*0.930*	*0.958*	0.956	0.901	0.933	0.960	0.724
39	*0.990*	*0.951*	*0.924*	*0.956*	0.915	0.951	0.921	0.985	0.735
40	*0.963*	*0.944*	*0.896*	*0.947*	0.962	0.973	0.985	0.956	0.833
41	*0.966*	*0.949*	*0.896*	*0.951*	0.968	0.946	0.980	0.955	0.858
42	*0.959*	*0.949*	*0.872*	*0.944*	0.965	0.962	0.997	0.955	0.852
43	*0.966*	*0.946*	*0.912*	*0.952*	0.965	0.958	0.967	0.955	0.809
44	*0.973*	*0.947*	*0.919*	*0.954*	0.950	0.930	0.927	0.962	0.762
45	*0.965*	*0.957*	*0.907*	*0.952*	0.969	0.951	0.966	0.959	0.857
46	*0.970*	*0.975*	*0.963*	*0.962*	0.957	0.875	0.884	0.961	0.616
47	*0.972*	*0.961*	*0.927*	*0.961*	0.963	0.906	0.917	0.953	0.822

48	*0.959*	*0.945*	*0.883*	*0.952*	0.970	0.963	0.990	0.951	0.857
49	*0.991*	*0.922*	*0.918*	*0.939*	0.913	0.965	0.920	0.985	0.661
50	*0.956*	*0.910*	*0.886*	*0.954*	0.968	0.960	0.997	0.966	0.743
51	*0.965*	*0.947*	*0.915*	*0.953*	0.961	0.924	0.946	0.956	0.731
52	*0.970*	*0.942*	*0.907*	*0.953*	0.964	0.956	0.976	0.952	0.830
53	*0.954*	*0.919*	*0.892*	*0.953*	0.980	0.974	0.981	0.964	0.828
54	*0.978*	*0.944*	*0.904*	*0.954*	0.959	0.927	0.971	0.960	0.745
55	*0.961*	*0.941*	*0.914*	*0.948*	0.961	0.924	0.942	0.962	0.638
56	*0.965*	*0.930*	*0.908*	*0.945*	0.952	0.955	0.935	0.976	0.716
57	*0.955*	*0.932*	*0.891*	*0.936*	0.959	0.930	0.957	0.968	0.646
58	*0.952*	*0.964*	*0.917*	*0.939*	0.969	0.873	0.913	0.950	0.627
59	*0.976*	*0.933*	*0.908*	*0.949*	0.931	0.971	0.945	0.986	0.691
60	*0.975*	*0.948*	*0.917*	*0.952*	0.943	0.923	0.914	0.964	0.719
61	*0.963*	*0.945*	*0.899*	*0.945*	0.970	0.964	0.968	0.968	0.864
62	*0.970*	*0.945*	*0.909*	*0.953*	0.947	0.928	0.935	0.967	0.639
**Mean**	***0.967***	***0.944***	***0.908***	***0.950***	**0.959**	**0.941**	**0.953**	**0.962**	**0.762**
**Std. Dev.**	***0.010***	***0.015***	***0.020***	***0.006***	**0.014**	**0.027**	**0.029**	**0.010**	**0.081**
**Max.**	***0.994***	***0.999***	***0.981***	***0.962***	**0.980**	**0.980**	**1.000**	**0.989**	**0.881**
**Min.**	***0.949***	***0.910***	***0.872***	***0.936***	**0.913**	**0.873**	**0.881**	**0.936**	**0.616**
