# Supplementary material for: A model of binding on DNA microarrays: understanding the combined effect of probe synthesis failure, cross-hybridization, DNA fragmentation and other experimental details of affymetrix arrays
Source: BMC Genomics. 2012 Dec 27;13:737. doi: 10.1186/1471-2164-13-737 (PMC3548757; doi:10.1186/1471-2164-13-737)

Supplementary Figure 1

For each Forward/Reverse probe pair the mean of the log (Forward/Reverse) was calculated across all 62 chips from the FMR1 design. Probes are binned by base composition with each bin corresponding to the excess of A over T nucleotides plus the excess of C over G nucleotides on the Forward strand. The numbers on the figure correspond to the number of distinct features that are observed in the bin. Observed (o) and expected means (*) are plotted for each bin.

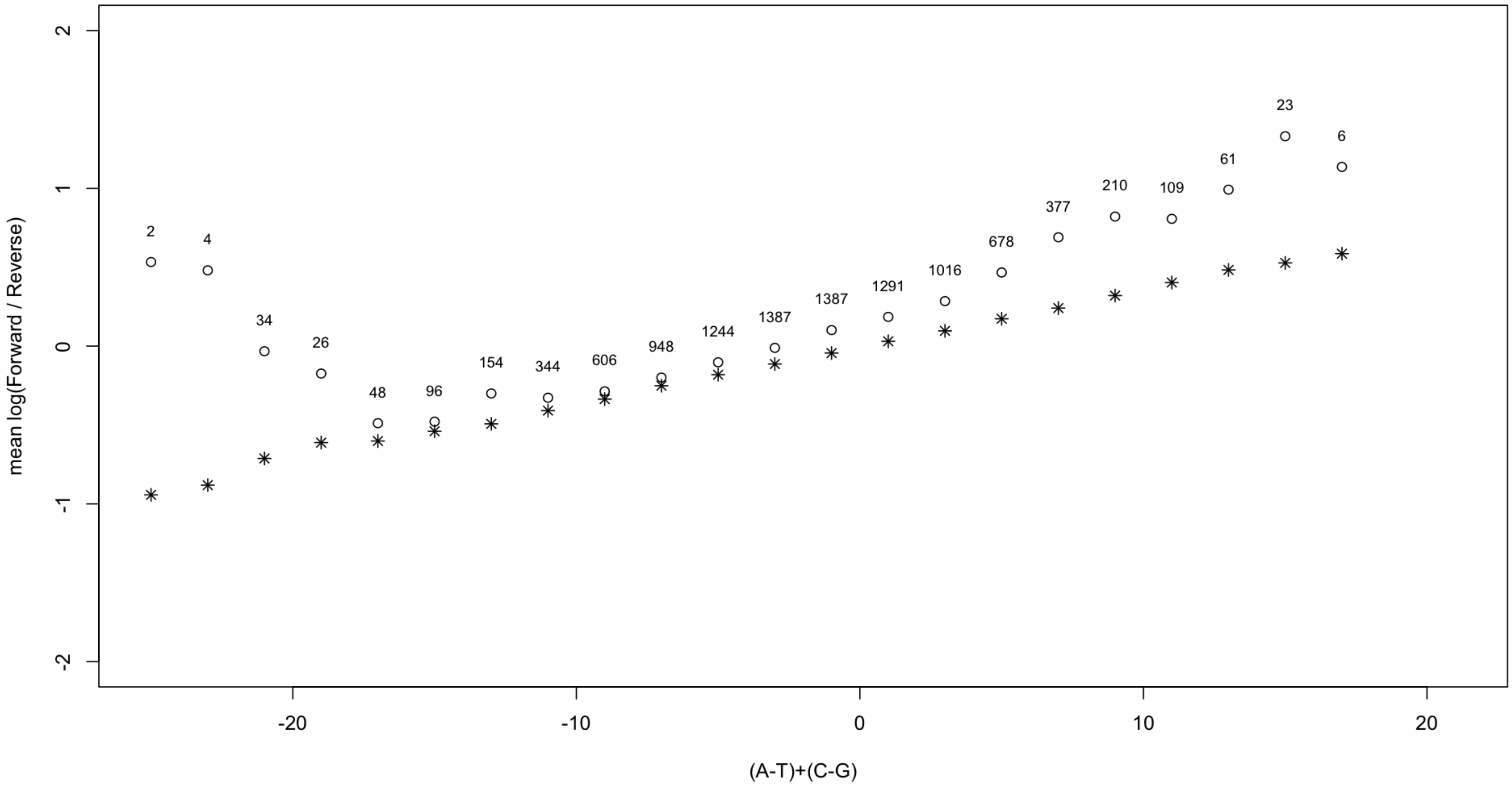

Supplement: Additional file 2 — Figure 1. For each Forward/Reverse probe pair the mean of the log (Forward/Reverse) was calculated across all 62 chips from the FMR1 design. Probes are binned by base composition with each bin corresponding to the excess of A over T nucleotides plus the excess of C over G nucleotides on the Forward strand. The numbers on the figure correspond to the number of distinct features that are observed in the bin. Observed (o) and expected means (*) are plotted for each bin. [file 1471-2164-13-737-S2.pdf]
